# Functional and comparative genomic characterization of biofilm formation in *Staphylococcus aureus*

**DOI:** 10.1016/j.bioflm.2025.100341

**Published:** 2025-12-09

**Authors:** Emily Rudolph, Shuxian Li, Broncio Aguilar-Sanjuan, Seungwon Ko, Priyanshu S. Raikwar, Carolin M. Kobras, Serena Bettoni, Samuel K. Sheppard, Maisem Laabei

**Affiliations:** a Department of Life Sciences, University of Bath, Bath, BA2 7AY, UK; b Ineos Oxford Institute, Department of Biology, University of Oxford, Oxford, OX1 3SZ, UK; c Sir William Dunn School of Pathology, University of Oxford, Oxford, OX3 1RE, UK; d School of Cellular and Molecular Medicine, University of Bristol, Bristol, BS8 1TD, UK

**Keywords:** Biofilm mechanisms, *Staphylococcus aureus*, Genome-wide association study, Pathogenicity

## Abstract

Biofilms are structured communities of bacterial cells enclosed in a self-produced extracellular matrix. In the pathogen *Staphylococcus aureus,* this can enhance resistance to antibiotics and immune responses, contributing significantly to chronic infections associated with medical devices. The underlying mechanisms include the production of polysaccharide intercellular adhesin (PIA), encoded by the *icaADBC* operon, and surface proteins that mediate adhesion. However, it has been challenging to translate *in vitro* understanding to explain the molecular mechanisms governing biofilm formation *in vivo*. Here we combined functional and comparative genomics approaches to investigate genetic factors influencing biofilm formation in isolates belonging to the clinically important ST-8 clonal complex (CC8). Phenotypic and genomic screening of a closely related strain cohort (MRSA USA300 isolates) revealed considerable variability in biofilm formation. Genome-wide association studies (GWAS) identified several genes and polymorphisms linked to biofilm development. These included known biofilm genes and compensatory mutations that restored wild-type biofilm levels in hyper-biofilm forming mucoid isolates. Finally, contextualizing CC8 genomes within diverse *S. aureus* populations revealed the natural occurrence of biofilm-associated genomic variation as well as evidence for the conservation of the *ica* loci in CC8. This offers insight into the mechanisms and microevolutionary events that give rise to clinically relevant staphylococcal infections.

## 1 Introduction

Bacterial biofilms are multicellular communities embedded within a self-produced matrix consisting of polysaccharides, proteins, extracellular DNA (eDNA), and lipids [1]. The formation of biofilms provides several advantages for bacteria including protection from antibiotic therapy and immune defences [2,3]. For example, biofilms can tolerate up to 1000 times the minimum inhibitory concentration of antibiotics of genetically identical planktonic cells [2]. Because of this enhanced survival strategy, biofilms are commonly associated with chronic infections [[4], [5], [6]].

*Staphylococcus aureus* is a major opportunistic and multi drug-resistant pathogen that causes a myriad of healthcare and community-associated infections [7]. In addition, *S. aureus* is a leading cause of implanted medical device infection as most clinically isolated *S. aureus* strains can form biofilms [8,9]. A key feature of staphylococcal biofilms is the production of the polysaccharide intercellular adhesin (PIA) or polymeric N-acetyl-glucosamine (PNAG) formed from the products of four genes, *icaA*, *icaD*, *icaB* and *icaC.* The separately transcribed *icaR* gene, located upstream of the *icaADBC* operon, encodes a DNA-binding protein that represses transcription of the *icaADBC* operon and plays a significant role in the environmental regulation of the *ica* operon [10]. Biofilm formation is also possible in *S. aureus*, independent of *ica* genes. This requires global virulence regulators including SarA and Agr and is dominated by the expression of cell wall anchored LPxTG-containing surface proteins such as fibronectin binding proteins A and B (FnBPA/B), biofilm associated protein (Bap) and *S. aureus* surface protein G (SasG) [8,[10], [11], [12]].

*In vitro,* mildly acidic conditions promote proteinaceous biofilm formation, independent of *ica* activity. This is commonly achieved by supplementing growth media with glucose [13]. Furthermore, studies comparing biofilm formation among methicillin-susceptible *S*. *aureus* (MSSA) and methicillin-resistant *S. aureus* (MRSA) isolates from device related infections revealed that MSSA exhibited enhanced biofilm formation under elevated NaCl conditions, linked to PIA production [14]. This contrasts with MRSA grown in media supplemented with NaCl or glucose, where PIA was not produced and *ica-*independent biofilm formation was mediated through the production of surface adhesins [14].

Despite the central role biofilms play in chronic infections, it has been challenging to link *in vivo* phenotype variation to infections in natural populations. This is largely because of incomplete understanding of the molecular mechanisms governing biofilm formation. To improve understanding of the genetics underlying biofilm formation we conducted detailed genotype and phenotype screening using a cohort of genetically related MRSA USA300 isolates which represent a subset of the ST-8 clonal complex. We showed that biofilm formation is highly variable in natural *S. aureus* populations and, employing genome-wide association study (GWAS) approaches, identified genes and mutations underlying this variation. Extending this approach to mucoid MRSA isolates, we identified mutations that promote hyper-biofilm-forming phenotypes and the compensatory mutations that revert this back to the wild-type phenotype. Finally, contextualizing isolates among natural *S. aureus* genetic variation, we found evidence of conservation of *ica* loci in the ST-8 clonal complex. Identifying the genetic drivers of biofilm formation in *S. aureus* has important implications for treating some of the most persistent bacterial infections.

## 2 Materials and methods

### 2.1 Bacterial culture, biofilm and minimum inhibitory concentration (MIC) assays

*S. aureus* clinical isolates (Suppl. Table 1) were plated on tryptic soy agar (TSA) (Merck; Cat. No: 22092) plates whereas *S. aureus* mutants containing the mariner *bursa aurealis* transposon obtained from Nebraska Transposon Mutant Library (NTML) [15] were plated onto TSA containing erythromycin (5 μg/mL). Confirmation of specific NTML mutants was achieved using colony PCR, gene-specific primers (Merck) (Suppl. Table 1) and Phusion HF master mix (ThermoFisher; Cat. No: F531S) according to manufacturer's instructions.

Congo red agar was prepared using brain heart infusion (BHI) agar (BD; Cat. No: 241830) supplemented with 3.6 % sucrose (Merck; Cat. No: S7903), 0.5 % glucose (Merck; Cat. No: G8270) and 0.08 % congo red (Merck; Cat. No: 234610). Congo red (Merck) was prepared in ddH_2_O first, filter sterilized and then added to molten agar, cooled to approximately 55 °C. Culture plates were incubated for 18–24 h at 37 °C following which single colonies were inoculated into 3 mL tryptic soy broth (TSB) (Merck; Cat. No: T8907) in 15 mL plastic tubes and grown overnight at 37 °C with 180 rpm shaking. The overnight cultures were diluted 1:200 into fresh TSB supplemented with glucose (TSB-G) either at 1 % for clinical isolates or 2 % for NTML mutants or 1 % NaCl (Merck; Cat. No: S9888) (TSB-NaCl). Based on our observations wild-type JE2 and the NTML Tn mutants formed more robust biofilms using 2 % vs 1 % TSB-G (data not shown). Individual bacterial strains were added in triplicate to a final volume of 200 μL in a 96-well Nunc Maxisorb plate (SLS; Cat. No: 442404) and incubated at for 24 h at 37 °C.

Semi-quantitative measurements of biofilm formation were determined using the method of Christensen et al. [16]. Following incubation, plates were washed vigorously four times with ddH_2_O and then stained with 250 μL of 0.5 % crystal violet (Merck; Cat. No: C0775), left for 30 min at room temperature. After staining, the plates underwent four additional washes with ddH_2_O and were then resuspended in 200 μL of 7 % acetic acid (Merck; Cat. No: 695092). The absorbance was measured at 595 nm using a Sunrise absorbance microplate reader (Tecan; Cat. No: 30133446). To control for day-to-day variability, we included a previously described control strain, TW20, that represents a biofilm-positive phenotype that forms consistent biofilms [17]. MRSA isolate TW20, representing the clonal complex 239 lineage, was included on each plate and percentage biofilm formation was normalised against this strain and wells containing no bacteria. Biofilm assays were performed using three technical repeats and repeated three times.

To determine minimum inhibitory concentration (MIC), overnight cultures of strain LAC (USA300; ST-8) [18] and USFL039 (USA300; ST-8) were grown in 2 mL of Mueller-Hinton broth (MHB) (Oxoid; Cat. No: 90922). Optical densities (OD_600_) of overnight cultures were measured using a spectrophotometer (Cole Palmer; Cat. No: Sp-200) and isolates were normalised in MHB to OD 0.1, then diluted 1:20 in MHB broth. For each antibiotic, (vancomycin (Merck; Cat. No: V2002), teicoplanin (Merck; Cat. No: T0578), polymyxin B (Merck; Cat. No: P4932), colistin (Merck; Cat. No: C4461), imipenem (Merck; Cat. No: I0160), nisin (Merck; Cat. No: N5764) and cefaclor (Merck; Cat. No: C6895), stock solutions were diluted in MHB. For daptomycin (ThermoFisher; Cat. No: 461371000) both culture and antibiotic dilutions were done in MHB with 50 mM CaCl_2_ (Merck; Cat. No: C1016). Each antibiotic was added to the 96 well plate (ThermoFisher; Cat. No: 10265362) in doubling concentrations, with a control well receiving no antibiotic. Plates were incubated at 37 °C overnight for 18 h. Vancomycin, teicoplanin and daptomycin were tested at 0.125–8 μg/mL; polymyxin B, colistin and nisin were tested at 8–512 μg/mL; imipenem and cefaclor were tested at 1–64 μg/mL. The MIC was determined as the lowest concentration that completely inhibited bacterial growth. Experiments were performed using two technical repeats and repeated three times.

### 2.2 Biofilm dispersal and antibiotic challenge

Following maturation of biofilms for 24 h as described above, plates were taken out and non-adherent cells and supernatant were removed by aspiration and biofilms further washed three times in ddH_2_O. For biofilm dispersal, compounds targeting extracellular DNA (DNase I (15 μg/mL); ThermoFisher; Cat. No: 10694233), proteins (proteinase K (0.5 mg/mL); Merck; Cat. No: P2308) or polysaccharides (sodium (meta)periodate (prepared in ddH_2_O; 20 mM); Merck; Cat. No: S1878) were added to biofilm containing wells for 2 h at 37 °C. After incubation, supernatants were removed and biofilms washed three times in ddH_2_O. Remaining biofilm biomass was quantified using the crystal violet method as described above. Experiments were performed using three technical repeats and repeated three times.

For antibiotic challenge antibiotic stock solutions were prepared in either TSB-G or TSB-NaCl. Vancomycin was made to a final concentration of 32 μg/mL and imipenem was 128 μg/mL. Antibiotics were serially diluted and added to mature (24 h) biofilms, with a control well receiving only TSB-G or TSB-NaCl. Replica plates were prepared to analyze biofilm formation following 24 h antibiotic challenge. Biofilm biomass reduction was analysed using the crystal violet method as described above. To examine metabolically active cells within the biofilm 2,3,5-triphenyl-tetrazolium chloride (TTC) (Merck; Cat. No: T8877) at 0.05 % (w/v) prepared in either TSB-G or TSB-NaCl was used. Following aspiration and washing of wells three times in ddH_2_O, 200 μL of TTC was added to biofilm wells and the plates were incubated for a further 20 h at 37 °C. Supernatant was removed and wells washed three times in ddH_2_O, following which the TTC crystals were resuspended in methanol (Merck; Cat. No: 34860). The absorbance was measured at 500 nm using a Sunrise absorbance microplate reader. Experiments were performed using three technical repeats and repeated three times.

### 2.3 Confocal microscopy

Biofilms were allowed to form for 12 h under conditions noted above with the modification of using sterile 96-well flat-bottomed (optically clear) black microtiter plate (ThermoFisher; Cat. No: 165305) conducive for confocal microscopy examination. Plates were removed from the incubator and non-adherent cells and supernatant removed by aspiration and wells washed three times in ddH_2_O. Following steps were performed in dark settings to preserve the fluorescence of dyes. A live/dead imaging solution was prepared by adding 2.5 μL of SYTO 9 and 15 μL Propidium Iodide (ThermoFisher; Live/Dead BacLight; Cat. No: L7012) in 10 mL sterile PBS (Oxoid; Cat. No: BR0014G). For the polysaccharide staining, a 2.5 μg/mL solution of wheat germ agglutinin (WGA) (Invitrogen; Cat. No: 11570806) was made in 10 mL PBS. In the black 96-well plate, each well received 200 μL of the Live/Dead solution or WGA solution and was then incubated for 30 min at room temperature (20 °C). After incubation, the stains were removed by pipetting, and 100 μL of PBS with no stain was added to each well. The plates were excited at 500 nm for both SYTO 9 and WGA and 575 nm for PI on a Zeiss Celldiscoverer 7 using Zeiss Zen Software (Version 3.10). Images were combined using the composite function of Fiji (Version 2.14.0, Java 1.8.0) and WGA samples were color adjusted to cyan for easier visibility and distinction from Syto 9. Relative fluorescence was also taken on a FLUOstar Omega microplate reader (BMG Labtech; Cat. No: 415-101-AFL) using the above wavelengths. Experiments were performed using two technical repeats and repeated three times.

### 2.4 Stability and relative fitness costs associated with hyper biofilm

Overnight cultures of 8 independent USFL039 isolates and one LAC (control) were made in 2 mL of TSB and grown for 18 h shaking at 180 rpm at 37 °C. The following day, cultures were (i) serially diluted 1:1000 and added to fresh TSB and grown for 18 h shaking at 180 rpm at 37 °C and (ii) diluted to 10^−6^ and 100 μL was plated on congo red agar and grown at 37 °C overnight. Congo red plates were analysed for changes in mucoid phenotype. This process was repeated for several days, alternating between plated colonies and liquid cultures. To test the biofilm forming capacity of strains, liquid cultures were collected, and isolates were normalised to OD_600nm_ 0.1, then diluted 1:20 in TSB broth. 10 μL of this culture was added to 190 μL of either TSB-G or TSB-NaCl and biofilms were left to mature for 24 h. Biofilms were examined using the crystal violet method as described above. Experiments were performed using three technical repeats and repeated three times.

The relative fitness costs associated with mucoid phenotype was examined by competing USFL039 against USFL039 non-mucoid. Overnight cultures were gently sonicated (45 s) and diluted to a concentration of 10^−4^ bacteria and mixed 1:1 in 3 mL in TSB-G. Separate tubes were used for each time point (4 h, 8 h and 24 h). At specific time points, cultures were gently sonicated, serially diluted in PBS and plated in triplicate on congo red agar for CFU enumeration. The fitness of the strain was defined as the reproductive success of the population, expressed as the natural logarithm of the ratio of the final and initial cell densities of the culture using the formula devised by Sanders et al. [19]; M_t_ = ln[(n_t_/m_t_)/(n_t-1_/m_t-1_)^1/gen^] where n_t_ and m_t_ are the amount of non-mucoid and mucoid cells at a given time-point t, while n_t-1_ and m_t-1_ represent the amount of non-mucoid and mucoid cells at the preceding time-point. *ln* is the natural logarithm (logarithm to the base *e*). The quotient of the ratios was standardized with the exponent 1/generation, assuming that cell numbers calculated at 24 h represents 17 generations [20]. The relative bacterial fitness for a given time was calculated as *fit*_*t*_ = 1+M_*t*_. If the non-mucoid isolate is fitter than the mucoid isolate the fitness value is bigger than 1. If there is no difference in fitness between the isolates, the value equals 1. If the non-mucoid isolate has a reduced fitness compared to the mucoid isolate the value is lower than 1.

### 2.5 Whole genome sequencing and comparative genomics

A total of 134 USA300 *S. aureus* clinical isolate genomes were analysed. These were derived from asymptomatic carriage, skin and soft tissue infection or bacteraemia [21,22]. Using a USA300 reference genome (FPR33757) we aligned the genomes of the clinical isolates using SNIPPY v4.6.0 (https://github.com/tseemann/snippy) on default settings and single nucleotide polymorphisms (SNPs) were identified. Core SNPs were concatenated using SNIPPY-CORE and SNP identities were compared based on their positions. To disregard sites that displayed monomorphism across the reference and clinical strains we employed the ‘-gtr’ flag. The alignment file was processed using GUBBINS v3.1.0 (https://github.com/nickjcroucher/gubbins) to minimise the impact of horizontal gene transfer on phylogenetic reconstruction. SNP_SITES v2.0.0 (https://github.com/sanger-pathogens/snp-sites) was used to extract SNP sites from prepared FASTA files. Employing a generalized time-reversible model we constructed a maximum likelihood phylogenetic tree using FastTree v2.1.11 (https://github.com/PavelTorgashov/FastTree).

The 8 independent cultures of USFL039 m were sequenced after the stability test to identify genes associated with the resolution of hyper biofilm formation. Genomic DNA was extracted using the QIAamp DNA Mini Kit (Qiagen, Germany) following the manufacturer's protocol, with the addition of 1.5 μg μL lysostaphin (Merck; Cat. No: L7386) and 2 μg mL lysozyme (Merck; Cat. No: L6876)) to enhance cell lysis. Sequencing was performed on an Illumina MiSeq platform. Libraries were prepared using Nextera XT kits (v2), and paired-end 250 bp reads were generated using the MiSeq run kit (v2). Assemblies were produced de novo with SPAdes (v3.3.0) [23]. Using the USFL039 genome as a reference we aligned the genomes of the USFL039nm 1–8 variants using SNIPPY v4.6.0 (https://github.com/tseemann/snippy) on default settings and single nucleotide polymorphisms (SNPs) were identified.

Whole genome assemblies were annotated using Prokka (version 1.12) [24] and annotated assemblies were processed by the PIRATE pan-genome pipeline (version 1.0.4) [25] to identify clusters of orthologous genes [26]. Components of the core genome were defined as genes present in more than 95 % of the isolates.

Genome-wide association analysis (GWAS) was performed on the genomes of 134 *S*. *aureus* USA300 isolates to identify genetic variants associated with biofilm formation. Variable-length *k*-mers were extracted from the genome assemblies and collapsed into unitigs. Pyseer v1.3.6 [27] was used to apply an elastic net model to determine the association between *k*-mers and biofilm formation. In addition, a lasso regression model was used and the default correlation filter of 25 % employed to reduce redundancy among highly correlated variants [27]. Population structure and relatedness among isolates were accounted for by incorporating a genetic relatedness matrix (GRM) derived from pairwise phylogenetic distances. The GRM was implemented within pyseer's linear mixed model (LMM) framework to control for lineage effects. This correction adjusts test statistics and associated *p*-values to account for shared ancestry and reduce confounding by clonal background. Following phylogenetic correction, the adjusted *p*-values were examined to identify variants significantly associated with biofilm formation. For functional interpretation, significant unitigs were mapped to the *S. aureus* USA300_FPR3757 complete reference genome using BWA-MEM v0.7.17 to infer their genomic context and potential gene function.

### 2.6 Genomic analysis of ica loci in natural *S. aureus* populations

To investigate the genetic variation across *ica* loci in natural populations, 134 USA300 isolate genomes [21,22] from this study were compared to a separate published dataset of 1991 UK isolates, sampled from clinical infections and asymptomatic carriage (BioProject PRJNA690682) [28]. For both datasets, genomes were assembled from paired reads using shovill version 1.1.0, with SPAdes as the default assembler (https://github.com/tseemann/shovill). Contiguous sequences (contigs) that were shorter than 500 bp were removed after assembly. A Grapetree was created to visualise the UK isolate dataset [29]. For this, clonal complexes (CCs) were defined to include sequence types (STs) with four or more allelic matches to the central ST for complexes CC1, CC5, CC8, CC15, CC22, CC30, CC45, CC93, CC93, and CC121, unless they were closer to another listed central ST. Allelic variation was quantified across the genome by BLAST comparison to archived loci in PubMLST.org [30]. A gene was deemed present where there was a nucleotide sequence identify match ≥90 % over ≥90 % of the gene length, and the number of unique alleles at each locus was determined [31,32]. For fine-grained nucleotide level analysis, genomes were screened using BLAST [33] and *ica* loci were identified, comprising the *icaR* and *icaADBC* genes and the *icaR_A* intergenic region. Sequences identified with BLAST were aligned using MAFFT version 7.525 [34] to reference strain USA300_FPR3757. Genetic variation across the *ica* loci was quantified in the USA300 and UK datasets, using GeneScanner (https://github.com/Sheppard-Lab/GeneScanner, [35]). Briefly, GeneScanner identifies the frequency of synonymous and non-synonymous mutations, stop codons, insertions, and deletions across aligned sequences. It applies two quality filters relative to a reference sequence, requiring at least 80 % ungapped coverage and 80 % pairwise identity. In nucleotide analysis mode, the program scans each alignment codon by codon while maintaining the correct reading frame, comparing translated codons to the reference to distinguish synonymous from non-synonymous changes. All mutation data are then compiled into a spreadsheet. The results were visualised for comparison using custom python scripts. The identified sequence variation was further manually compared to previously reported mutational sites and any overlap with regulatory elements in the *icaR_A* intergenic region.

## 3 Results

### 3.1 Biofilm formation varies among genetically related clinical *S. aureus* isolates

**Fig. 1:**
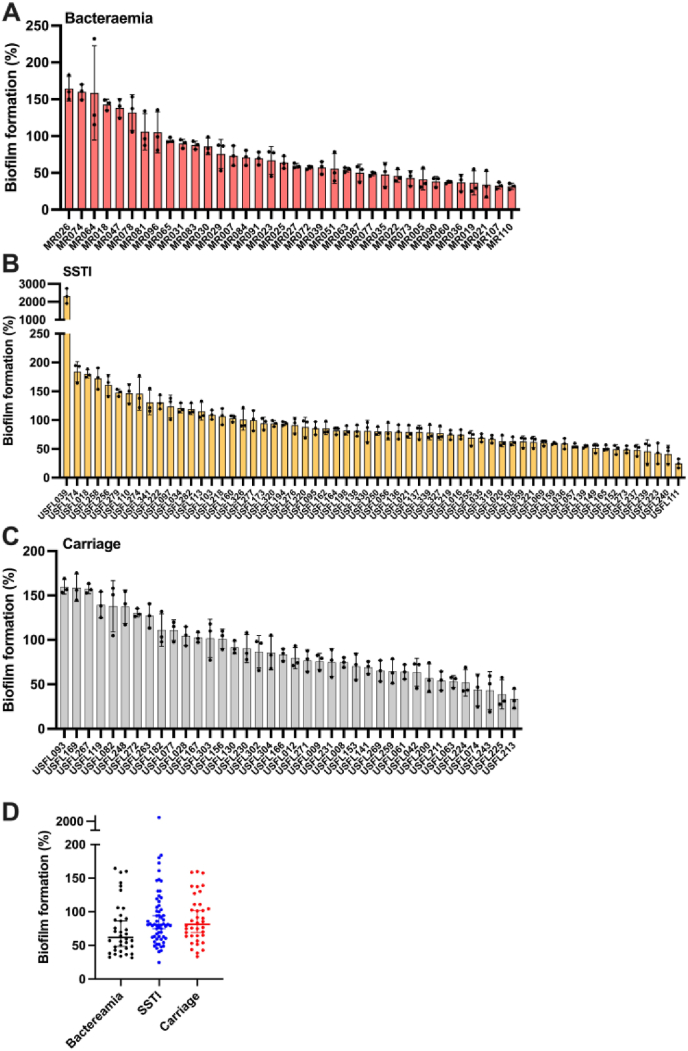
**Biofilm formation of 134 clinical MRSA CC8 *S. aureus* strains.** Biofilm development under static conditions was examined using the crystal violet assay. **A)** Bloodstream isolates (n = 36), **B)** skin and soft tissue infection (SSTI) isolates (n = 60) and **C)** isolates derived from asymptomatic carriage (n = 38) were examined and percentage biofilm formation compared to a control MRSA isolate, TW20 (designated 100 %). Three technical and three biological repeats were used with columns and error bars displaying the mean and standard deviation. **D)** Comparison of biofilm formation from three clinical sources where each dot represents the average biofilm formation of each isolate. A one-way ANOVA with Tukey multiple comparison test showed no significant difference in biofilm formation between groups. (For interpretation of the references to colour in this figure legend, the reader is referred to the Web version of this article.)

Variation in biofilm formation was assessed within a collection of 134 CC8 (USA300, CA-MRSA, SCC*mec* type IV) *S. aureus* isolates originating from bloodstream infections (n = 36) (Fig. 1a), skin and soft tissue infections (n = 60) (Fig. 1b) or from asymptomatic carriage of the skin or nose (n = 38) (Fig. 1c). Despite the close genetic relatedness, biofilm forming capacity varied across the cohort of strains; 23 strains were considered low biofilm forming isolates with biofilm production less than 50 % of the control strain TW20, while a further 23 strains were considered high biofilm formers, generating biofilms more than 125 % of the control strain. One strain, USFL039 (SSTI isolate) formed biofilm greater than 2000 % of TW20, and therefore exhibited hyper biofilm formation, defined in our study as greater than 10-fold biomass compared to the control strain. To explore the potential correlation between biofilm development and infection severity, we compared the relative biofilm formation across the three different cohorts (Fig. 1d).

**Fig. 2:**
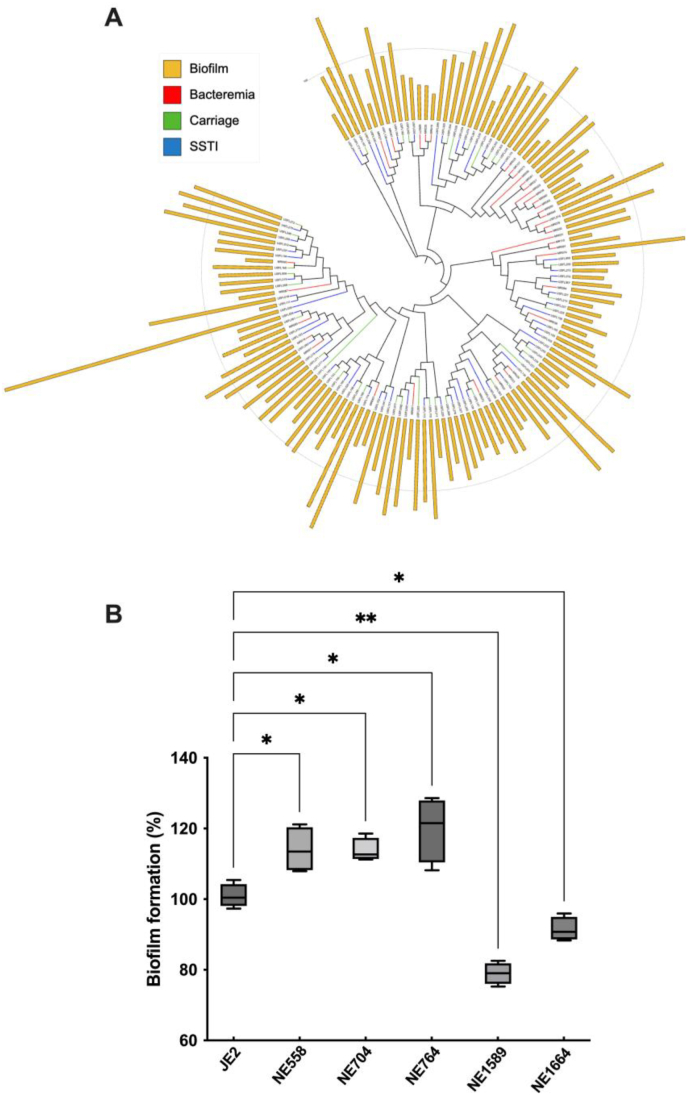
**Maximum likelihood tree displaying biofilm formation and functional validation of biofilm associated genes derived from GWAS. A)** A SNP-based maximum likelihood tree mapping biofilm formation and infection source across the collection of isolates. Branch colours describe different disease origins; red = bacteraemia; green = carriage; blue = SSTI. External bars represent normalised biofilm formation; USFL039 biofilm formation is displayed not to scale. **B)** Transposon insertion mutants were assessed for biofilm activity compared to wildtype JE2. Three biological repeats were included. Statistical differences were calculated by one-way ANOVA. ∗*p* < 0.05, ∗∗*p* < 0.01. (For interpretation of the references to colour in this figure legend, the reader is referred to the Web version of this article.)

Although bloodstream isolates had a trend towards forming weaker biofilms compared to carriage isolates it was not statistically significant (*p* = 0.11). Furthermore, mapping biofilm formation across a core genome *S. aureus* maximum likelihood tree revealed no significant clustering biofilm forming lineages (Fig. 2a).

### 3.2 Genome-wide association analysis reveals biofilm-linked loci

K-mers associated with enhanced biofilm formation, in the GWAS, were mapped to the *S. aureus* pangenome. The elastic net model, employing regularized linear regression, enabled simultaneous evaluation of the association between all genetic variants and the phenotype (Suppl. Fig. 1). A quantile-quantile plot of observed *p*-values in the elastic net model indicated the presence of lineage effects suggesting that variation within several loci may be responsible for altered biofilm formation. To distil the causal variants to a single locus with the most substantial effect on biofilm formation, a lasso regression model was employed. A substantial number of unique *k*-mers were identified as significantly associated with biofilm formation aligning with 32 genes in the *S. aureus* pangenome. These genes belong to functional categories including transport and binding proteins, DNA replication, repair and recombination, cell envelope biogenesis, carbohydrate transport and metabolism, as well as genes involved in virulence and hypothetical proteins (Suppl. Table 2).

Validation of bacterial GWAS results is increasingly common to improve the robustness of analyses [[36], [37], [38], [39]]. In order to validate the GWAS-identified *k*-mers linked to biofilm formation, we made use of the NTML [15]. Of the 32 genes identified in our GWAS, 25 had corresponding mutants available from the NTML and 5 were associated with a statistically significant difference in biofilm formation compared to the wildtype strain (JE2) (Fig. 2b). Transposon mutants NE1589 (*metQ1*, ABC transporter substrate-binding protein) and NE1664 (*arcC*, carbamate kinase) were found to have reduced biofilm formation while NE558 (*lukE*, leukotoxin E), NE704 (*araB*, l-ribulokinase) and NE764 (SAUSA300_1432, phiSLT ORF78-like protein) displayed enhanced biofilm activity; correct transposon insertion in the above genes was confirmed using PCR with gene-specific primers (Suppl. Fig. 2).

### 3.3 Phenotypic characteristics of hyper-biofilm formation in strain USFL039

**Fig. 3:**
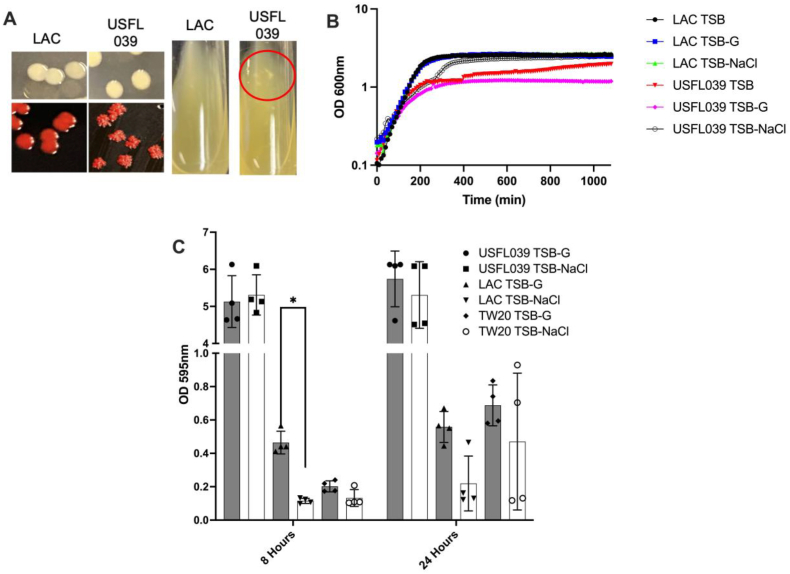
**Phenotypic characterisation of strain USFL039. A)** Strains LAC (control) and USFL039 were grown on TSA, congo red agar (CRA) or in liquid TSB culture. USFL039 growth on CRA and in liquid culture (red circle) displays biofilm and aggregative phenotypes respectively. **B)** Growth kinetics of LAC or USFL039 grown in either TSB, TSB-G or TSB-NaCl. Graphs displays average OD_600nm_ measurements of two biological replicates. **C)** Biofilm forming capacity of strains LAC, TW20 or USFL039 grown for either 8 or 24 h in TSB-G (grey bar) or TSB-NaCl (white bar). Each dot represents individual biological repeat with error bars displaying the SD. Statistical differences were calculated by two-way ANOVA. ∗*p* < 0.05. (For interpretation of the references to colour in this figure legend, the reader is referred to the Web version of this article.)

The biofilm phenotype was assessed to explain phenotypic anomalies using several approaches, including: (i) detailed analysis of USFL039 colony morphology, growth kinetics and the impact of environmental conditions; (ii) the influence of antibiotic resistance; (iii) microscopic variation among hyper-biofilm forming isolates. Strain USFL039, an MRSA strain isolated from SSTI, displayed a 20-fold increase in biofilm formation compared with control USA300 strain LAC (Fig. 1b). It aggregated in liquid culture and formed dry irregular shaped colonies on congo red agar (Fig. 3a). Furthermore, it grew slower in TSB and TSB supplemented with 1 % (w/v) glucose (TSB-G) or 1 % (w/v) NaCl (TSB-NaCl) (Fig. 3b) compared to the LAC strain. These features are similar to previously described hyper-mucoid *S. aureus* isolates that overexpress PIA/PNAG [20,40]. To help determine whether the hyper-biofilm phenotype was dependent on specific environmental conditions present in the growth media, we examined biofilm formation in both TSB-G and TSB-NaCl (Fig. 3c). There was no significant difference in the USFL039 biofilms when grown in the presence of glucose or NaCl however control strains LAC and TW20 tended to form larger biofilms in the presence of glucose rather than NaCl, with LAC having a significantly larger biofilm at 8 h in glucose (p = 0.0428).

**Fig. 4:**
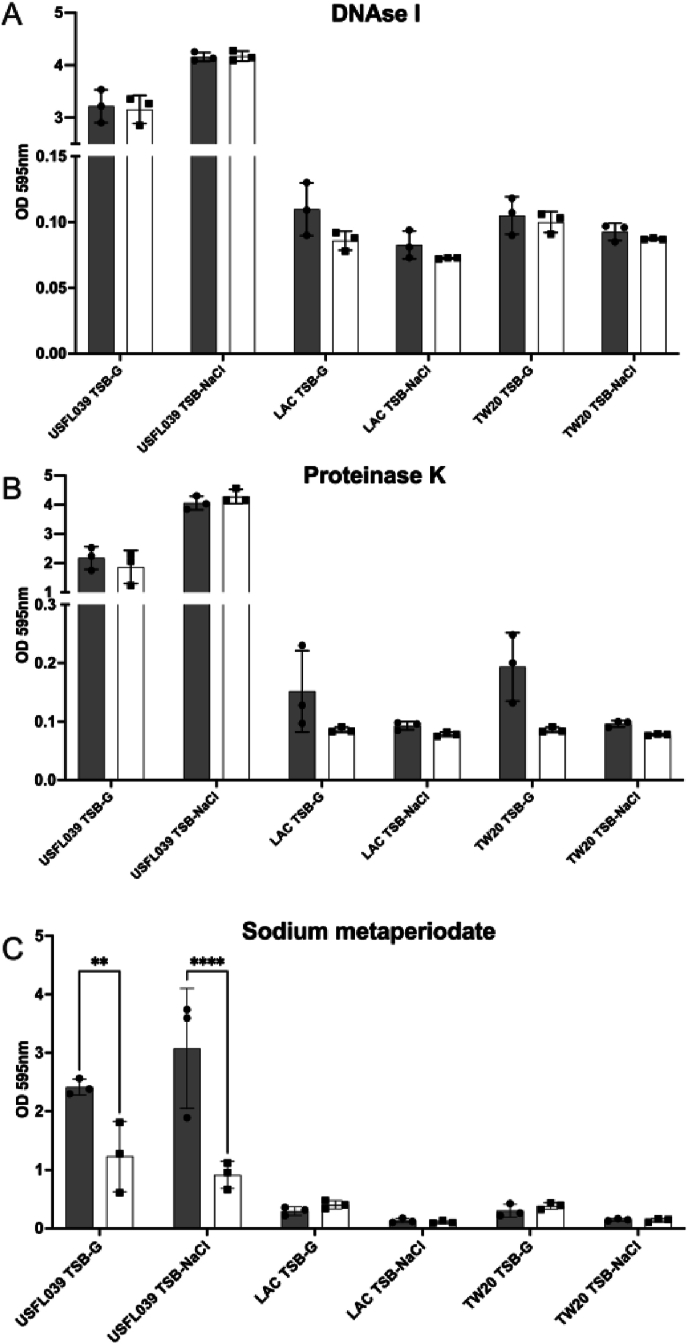
**Major biofilm component of strain USFL039.** Mature biofilms of USFL039, LAC and TW20 grown in TSB-G or TSB-NaCl were incubated with disrupting agents (white bars) **A)** DNase I (15 mg/mL) **B)** proteinase K (0.5 mg/mL) **C)** sodium metaperiodate (20 mM) for 2 h at 37 °C, after which remaining biofilms were stained. Controls (grey bars) were mature biofilms grown under identical conditions that received no challenge. Significance was determined through a 2-way ANOVA, where ∗∗ indicates a *p* value less than 0.01 and ∗∗∗∗ indicates a p value < 0.0001.

To determine the composition of the USFL039 biofilm matrix, we treated this strain with compounds/enzymes that target specific components of staphylococcal biofilms. DNAse I, proteinase K and sodium metaperiodate were used to investigate the importance of DNA, proteins and polysaccharides in hyper-biofilm formation respectively. DNAse treatment had no significant impact on biofilm development on USFL039 or control strains LAC and TW20 grown in either TSB-G or TSB-NaCl (Fig. 4a). Proteinase K treatment resulted in lower biofilm formation in both strain LAC and TW20 grown in TSB-G but this was not significant; USFL039 grown in TSB-G or TSB-NaCl was not impacted by proteinase K treatment (Fig. 4b). Sodium metaperiodate treatment causes a significant decrease in USFL039 biofilm under both TSB-G (*p* = 0.0026) and TSB-NaCl (*p* < 0.0001) (Fig. 4c) indicating that the major component driving hyper-biofilm formation under both conditions is the production of PIA.

To quantify how USFL039 hyper-biofilm production translated to increased antibiotic resistance, we examined the impact of long-term exposure of two clinically important antibiotics. Mature biofilms of USFL039, LAC and TW20 grown in TSB-G or TSB-NaCl, exposed to increasing concentrations of either vancomycin or imipenem, revealed no significant difference in biomass or metabolic activity (Suppl. Fig. 3A–D). However, both LAC and TW20 displayed decreased biofilm formation and metabolic activity with increasing concentration of antibiotic treatment. To note we tested the MIC of several different classes of antibiotics against USFL039 and LAC and observed no difference (Suppl. Table 3).

**Fig. 5:**
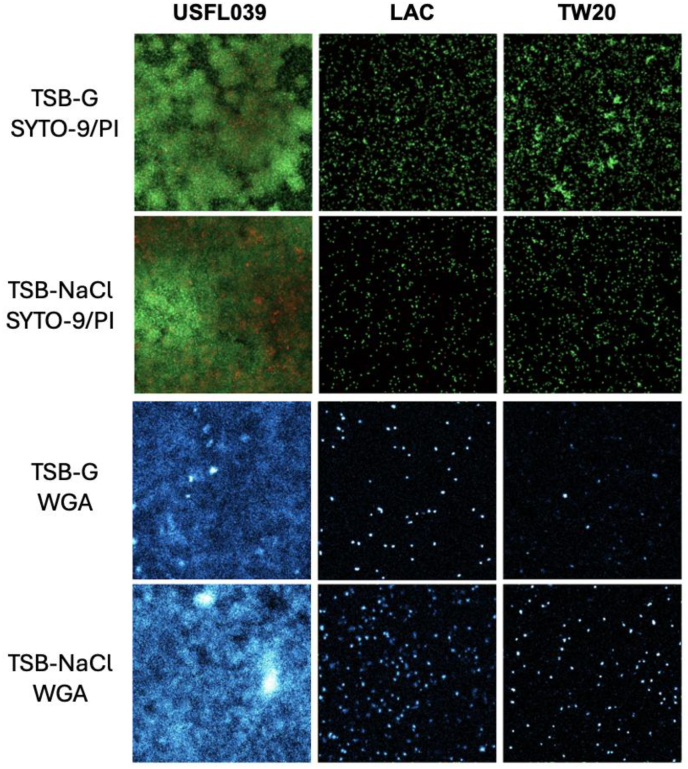
**PIA/PNAG straining and biofilm visualisation by confocal microscopy.** USFL039, LAC and TW20 biofilms were grown for 12 h in either TSB-G or TSB-NaCl. Live-dead staining was performed using SYTO9 and propidium iodide (PI); polysaccharide staining was performed using fluorescently labelled wheat germ agglutinin (WGA). The wells were excited at 500 nm for both SYTO9 and WGA and 575 nm for PI on a Zeiss Cell discoverer 7. Green indicates live cells stained by SYTO9, red indicates dead cells stained by PI, and cyan indicates polysaccharides produced by the *ica* operon stained by WGA. (For interpretation of the references to colour in this figure legend, the reader is referred to the Web version of this article.)

Finally, consistent with previous studies, wheat germ agglutinin (WGA) was used as an indicator of PIA/PNAG expression in staphylococcal biofilms [41,42]. Using fluorescently labelled WGA and confocal microscopy, we investigated the expression of PIA in USFL039 and control strains LAC and TW20 grown in either TSB-G or TSB-NaCl for 12 h. Biofilms formed after 12 h maturation (Suppl. Fig. 4a) and the ratio of live/dead staining, using SYTO9 and propidium iodide (PI) respectively, indicated that all strains under both conditions contained living cells. Interesting there was a significantly higher number of dead cells compared to live cells in USFL039 biofilms and more dead cells in biofilms grown in TSB-G (Suppl. Fig. 4b). Following WGA staining, USFL039 had significantly more PIA than LAC or TW20 when grown in TSB-G or in TSB-NaCl (Fig. 5, Suppl. Fig. 4c). No significant difference in PIA expression was observed when USFL039 was grown in TSB-G or TSB-NaCl, however there was significantly more PIA present in LAC (p = 0.0005) and TW20 (p < 0.0001) biofilms grown in the presence of NaCl.

### 3.4 Genotype characteristics of hyper-biofilm formation in strain USFL039 were linked to ica genes

To investigate the genetics associated with hyper-biofilm formation, we compared the genome sequences of USFL039 against the highly related, non-hyper biofilm, CC8 reference strain LAC. Single nucleotide polymorphisms, insertion and deletions were recorded (Suppl. Table 4). In total there were 33 non-synonymous SNPs, 7 frameshift events and 1 non-sense mutation occurring in the USFL039 genome, compared to strain LAC. In the context of hyper-biofilm formation, a frameshift mutation occurring in the *icaR* gene (TC > T at nucleotide position 314, coding for E105) resulted in a premature stop codon at position 120 and truncation of IcaR. Loss of *ica* regulation was the most likely cause of hyper-PIA production and the mucoid phenotype.

### 3.5 Hyper-biofilm phenotypes are rapidly lost *in vitro* suggesting a fitness cost

**Fig. 6:**
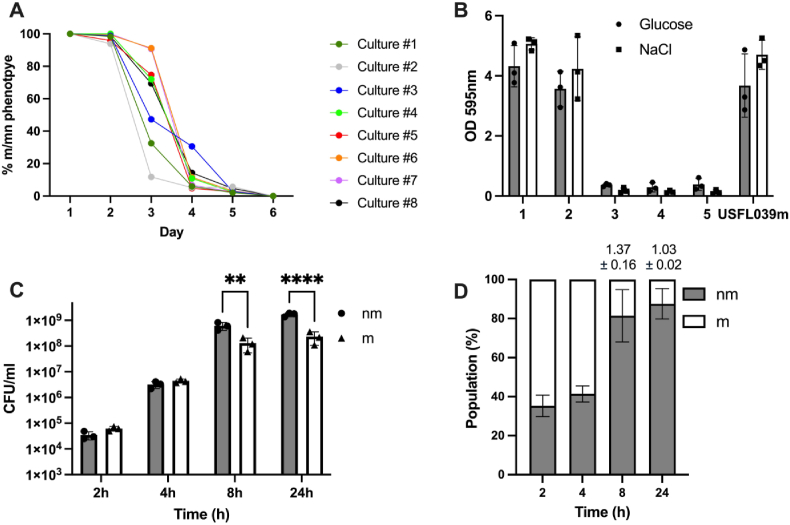
**Mucoid phenotype is rapidly lost *in vitro* suggesting a fitness cost. A)** Frequency of mucoid to non-mucoid phenotype reversion was calculated every 24 h following screening on CRA agar. **B)** Biofilm capacity of USFL039 m culture #2 after each passage day grown in TSB-G or TSB-NaCl. **C)** Co-culture competition experiments between USFL039nm and USFL039 m; mucoid phenotype was examined on CRA agar at time points 2, 4, 8 and 24 h and presented in CFU/ml. Each dot represents individual biological repeat with error bars displaying the SD. Statistical differences were calculated by two-way ANOVA. ∗∗*p* < 0.01; ∗∗*p* < 0.0001**. D)** Co-culture experiments presented in C are displayed as bars representing the value of each phenotype as percent of total population with fitness values indicated on top of the graph.

To examine the frequency of reversion of USFL039 mucoid (USFL039 m) to non-mucoid (USFL039nm) when grown under nutrient-rich laboratory conditions, we inoculated 8 independent cultures of USFL039 m in TSB-G and examined reversion of mucoid phenotype on CRA following passage every 24 h (Fig. 6a). We observed 100 % of colonies exhibiting mucoid phenotype after 2 days passage but this altered significantly on day 3 with frequency of non-mucoid phenotype ranging from 10 to 90 %. Non-mucoid phenotype was observed in 75–90 % of cultures by day 4, greater than 95 % by day 5 while all cultures were observed to have reverted to non-mucoid phenotype by day 6 (Fig. 6a). To confirm that the hyper biofilm phenotype was lost during *in vitro* serial passage we tested the biofilm capacity of USFL039 m culture #2 in TSB-G and TSB-NaCl; biofilm capacity and mucoid phenotype correlated whereby day 3 biofilm capacity was lost (Fig. 6b).

To determine if hyper-expression of PIA/PNAG confers a fitness cost, we performed competitive co-culture experiments with equivalent inoculum sizes of USFL039 m and USFL039 nm (culture #2). We examined shifts in the population over a 24 h period by assessing colony morphology on CRA. We observed that there does appear to be a significant growth advantage in the non-mucoid strain, particularly after 8 h and 24 h of growth competition (Fig. 6c–d).

Sequencing of the 8 independently generated non-mucoid USFL039 revertants revealed 6 acquired mutations in *ica* genes (Suppl. Table 5). Notably, we identified 3 frameshift mutations in *icaC*, a gene encoding a transmembrane protein essential for exporting polysaccharides to the cell surface [10]. Strain #1 and #8 harboured TTTA deletions while strain #2 acquired a TTTA insertion at the same position, introducing a premature stop codon and producing a truncated IcaC protein at position I286. Strain #5 contained a disruptive in-frame deletion between nucleotide position 564–575 of *icaC*, resulting in a truncated IcaC protein at position I192. Strain #7 acquired a non-synonymous mutation within *icaC* resulting in a G92R. Strain #4 presented with a frameshift mutation following deletion of ATTTAC at nucleotide positions 187–192 within *icaB*, a gene encoding a deacetylase that modifies mature PIA/PNAG by removing acetyl groups, resulting in a net positive charge and enhanced biofilm formation [43]. Interestingly strains #3 and #6 harboured no mutations within the *ica* operon; #3 contains a non-synonymous mutation within a hypothetical gene not currently associated with *ica* activity while genome comparisons between #6 and USFL039 m offered no genetic basis for reversion to nm phenotype.

### 3.6 Biofilm expression is modulated via a regulatory intergenic region in CC8 *S. aureus*

**Fig. 7:**
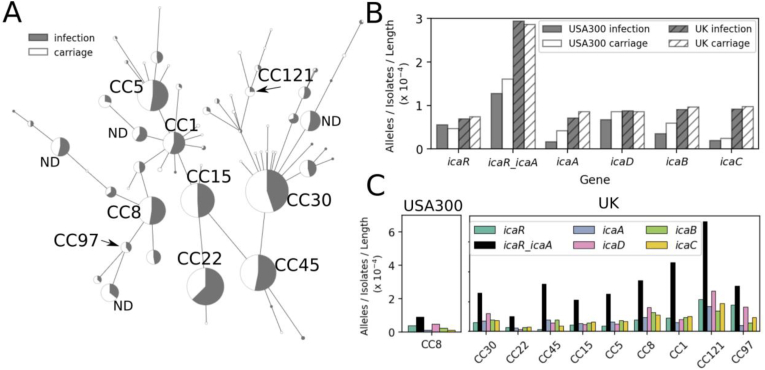
**The *ica* locus is present in most *S. aureus* isolates, with greatest variation in regulatory intergenic region. A)** Grapetree of 1991 *S. aureus* isolates, from carriage (white) and infection (grey). **B)** Number of unique alleles for *icaR*, *icaADBC* and the *icaR_A* intergenic region for the USA300 isolates used in this study (grey) and the more diverse UK dataset (striped). Frequencies are normalised to the number of isolates and sequence length. **C)** Number of unique alleles (as above) for each major clonal complex.

Genetic variation across the *ica* locus in the 134 USA300 *S. aureus* was compared with a UK dataset comprising 1991 *S. aureus* isolate genomes sampled from bacteraemia cases and asymptomatic carriage [28]. While the USA300 isolates all belonged to CC8, the second dataset comprised isolates from all major CCs, including CC8 (Fig. 7a). We found the *ica* locus, comprising *icaADBC*, the divergent *icaR* locus, and the *icaR_A* intergenic region, were present in all 134 USA300 isolates and most of the UK dataset (1975/1991). This is consistent with conservation of *ica* locus presence within genetically diverse natural *S. aureus* populations. The number of unique alleles for each *ica* gene, and the intergenic region, was quantified and normalised it to the size of the datasets and gene length. Reflecting the broader phylogeny within the UK isolates, all genes had more unique alleles in this dataset compared to the USA300 isolates (Fig. 7b) with the *icaR_A* intergenic region containing the most unique alleles, indicating greater variability. Interestingly, there was no difference in the number of unique alleles between isolates sampled from disease or carriage across both datasets. Comparison of the unique alleles for each clonal complex further highlighted the strong genetic conservation of the *ica* locus in the USA300 isolates, with more unique alleles observed for the CC8 of the UK dataset (Fig. 7c). Again, the *icaR_A* intergenic region, which contains the binding sites for several regulatory elements [44], was the most variable across all clonal complexes, indicating strain-level modulation of biofilm expression.

### 3.7 Population analysis of the ica locus suggests loss of mucoid phenotype in natural *S. aureus* isolates

**Fig. 8:**
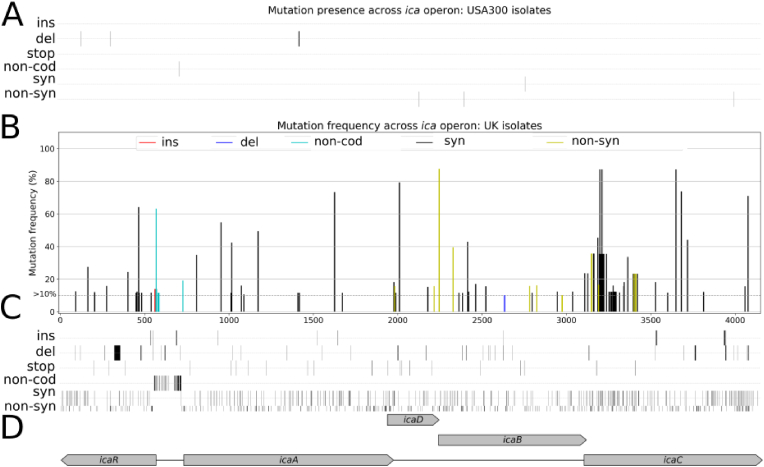
**Population analysis of the *ica* locus suggests loss of mucoid phenotype in natural *S. aureus* isolates. A)** Binary heatmap of mutations at each alignment position of the *ica* locus for the USA300 dataset. **B)***ica* locus mutations with frequencies over 10 % for the UK dataset, including insertions (red), deletions (blue), synonymous (black) and non-synonymous (gold) mutations and mutations in non-coding sequences (cyan). **C)** Binary heatmap of mutations <10 % at each alignment position of the *ica* locus for the UK dataset isolate datasets. **D)** Diagrammatic representation of the *ica* operon. (For interpretation of the references to colour in this figure legend, the reader is referred to the Web version of this article.)

Fine-grained mutational analysis of the isolates revealed distinct patterns of nucleotide alterations across the *ica* locus**.** In USA300 isolates, the *ica* locus was highly conserved, with low mutation frequencies (<5 %) and only 8 polymorphic sites in total (Fig. 8a). Notably, two frameshift mutations in *icaR* were identified: G313del in the hyper-biofilm isolate USFL039 and A136del in MR073. Both mutations resulted in premature stop codons, truncating the IcaR protein at 116 and 63 amino acids, respectively. These truncations have been previously reported in mucoid isolates from cystic fibrosis patients [45]. However, MR073 exhibited reduced biofilm formation, suggesting a rapid loss of the mucoid phenotype despite the presence of such mutations.

In contrast to the USA300 collection, the UK isolates showed extensive variability, with 725 polymorphic sites, including 10 non-synonymous mutations present at frequencies >10 %, primarily within *icaDBC* (Fig. 8b–c). Over 10 % of isolates carried a 1-nt insertion in the *icaR_A* intergenic region. Importantly, variations were also found in the Rob repressor binding sites, including modifications or complete loss of the TATTT motif, essential for Rob recognition and binding, indicating constitutive *icaADBC* expression in some isolates [45,46] (Fig. 8b–c). A single nucleotide deletion in *icaB*, observed in more than 10 % of isolates, led to a premature stop codon at M161, likely disrupting exopolysaccharide synthesis. Similarly, a frame shift at I63, which we previously associated with the loss of the mucoid phenotype *in vitro*, was also detected in 13 natural isolates, suggesting its functional significance in the real world. Further analysis identified TTTA insertions or deletions in *icaC* across 93 isolates, resulting in frameshifts, premature stop codons, and truncation of IcaC (Fig. 8b–c). These alterations are known to inactivate IcaC, leading to loss of PIA/PNAG production and the mucoid phenotype [20], consistent with observations in our USFL039 non-mucoid strains. Additionally, a substitution at IcaC G92 linked to mucoid loss *in vitro* was found in 27 *S. aureus* isolates. Collectively, these findings suggest that the mucoid phenotype is frequently lost in *S. aureus*, both *in vitro* and in real world clinical settings, likely due to a fitness cost.

## 4 Discussion

*S. aureus* biofilm formation is associated with increased antibiotic resistance, chronicity and recurrent infections. Therefore, understanding the molecular mechanisms governing biofilm development is an important step in developing effective therapeutic interventions. We employed a functional genomics approach combining phenotypic biofilm screening of 134 closely related USA300 clinical isolates with a *k*-*mer*-based GWAS to identify genetic factors associated with biofilm formation. All tested *S. aureus* clinical isolates could form biofilms, but the degree of formation was variable and not significantly associated with disease or carriage. Population-wide biofilm phenotypes and genes were compared with data from a genome-wide transposon library. Of the 32 biofilm-associated genes flagged in the GWAS, 25 had corresponding Tn mutants within the NTML strain library. Testing these, revealed 5 mutants that displayed significantly altered biofilm formation.

Of the genes linked to biofilm formation in both the GWAS and deletion library, *lukE* encodes a subunit of the bi-component, pore-forming leucocidin, LukED [47]. There is no known association between LukED and biofilm development in *S. aureus*, however staphylococcal toxins are known to have a role in biofilm development and dispersion. *S. aureus* alpha haemolysin has been shown to be important in cell-to-cell interactions required for initial biofilm development [48]. Beta toxins appear to form a nucleoprotein complex with extracellular DNA, providing a foundation for biofilm establishment [49]. The staphylococcal phenol-soluble modulin (PSM) class of peptide toxins are central in biofilm dispersion owing to their surfactant-like properties [50]. Ribulokinase B (*araB*), an enzyme involved in the arabinose catabolic pathway and carbamate kinase (*arcC*), an enzyme involved in the arginine deiminase pathway displayed enhanced and reduced biofilm formation respectively when disrupted. This is consistent with studies linking alterations in metabolism to differential biofilm formation [[51], [52], [53]], and the importance of the arginine deiminase operon in reduced PIA accumulation [54], and biofilm development.

Hyper-biofilm, mucoid forming *S. aureus* are rare among clinical isolates but provide a valuable opportunity to understand the mechanisms underlying biofilm phenotypes.

Experiments investigating the regulation of the *ica* locus have described a 5-nucleotide deletion (TATTT) within the *icaR*-*icaA* intergenic region which caused enhanced *ica* transcription that promoted a hyper-biofilm phenotype [55]. DNase I footprint analysis has revealed that deletion of this motif did not impact the affinity of IcaR to this region, indicating the existence of an additional repressor. A new repressor of the *ica*-locus, defined as the repressor of biofilms (*rob*), has been described that recognised the TATTT motif within intergenic region of the *ica* operon [46]. Furthermore, recent studies have identified hyper-PIA/PNAG, mucoid *S. aureus* isolated from respiratory samples of people with cystic fibrosis (pwCF) which all carried this 5 bp deletion (TATTT) in the intergenic region of the *ica* operon [40,56]. A subsequent study by the same group identified mucoid *S*. *aureus* isolates not housing the intergenic *ica* 5 bp deletion but found several mutations within the *icaR* gene resulting in truncated IcaR and associated hyper biofilm elaboration [45]. Through phenotypic, biochemical, microscopic and sequencing analysis we discovered in our study that USFL039 overproduces PIA and harbours a frameshift mutation in *icaR*, the repressor of the *icaADBC* operon. IcaR is a homodimer and member of the tetracycline repressor (TetR) family of transcriptional regulators which recognises a 42-nt intergenic region immediately upstream of the *icaA* gene [57]. USFL039 houses a mutation resulting in a truncated IcaR at position amino acid 120, 66 amino acids shorter than the full-length protein. Whole genome sequencing pointed to the mutation in *icaR* as the primary cause of PIA-mediated biofilm development.

Previous work has shown that expression of PIA protects against phagocytic killing [40]. However, due to the energetic costs associated with hyper biofilm formation, it is possible that mucoid strains would be less fit than non-mucoid strains. We observed that hyper-production of PIA is a phenotype that is rapidly lost following *in vitro* serial passage in nutrient-rich media. Co-culture competition experiments between rival mucoid and non-mucoid USFL039 stains indicated that mucoid strain was less fit than non-mucoid counterpart, exhibiting only 10 % of the population following 24 h growth.

Following isolation of non-mucoid USFL039 variants (Fig. 6a), we performed whole genome sequencing to identify genetic changes associated with reversion. Our analysis revealed frameshift mutations arising due to deletion (strains #1 and #8) or insertion (strain #2) of a 4-nucleotide repeat (TTTA) in the *icaC* gene, resulting in a truncated protein of 292 amino acids, 58 amino acids shorter than wildtype IcaC. Strain #5 harboured an in-frame deletion within *icaC* resulting in a truncated IcaC at position I192 while strain #7 presented with a non-synonymous mutation within *icaC* resulting in a G92R; substituting glycine with the larger, positively charged arginine can profoundly impact protein structure and function, resulting in altered protein stability, activity and/or interactions with other proteins with the net impact of reduced biofilm formation. Our data showed that 5/8 of non-mucoid USFL039 variants had mutations within *icaC*. IcaC is an integral membrane protein believed to contain 10 transmembrane helices and functions to link short PIA oligomers, synthesized by the IcaA and IcaD, into longer polymer chains and to transports these polymers to the bacterial surface [58]. Brooks et al. identified that a change in the number of TTTA repeats in *icaC* resulted in a variable, PIA-on-off phenotype [20]. We have not observed the mucoid phenotype to switch back on following subsequent passage.

The *icaB* gene encodes a deacetylase essential for retaining PNAG on the bacterial surface and for efficient biofilm formation. Previous studies have shown that a laboratory-generated *icaB* mutant of *S. aureus* produces significantly reduced levels of surface-associated PNAG [59]. Interestingly strain #4 contained a deletion within *icaB* that resulted in truncated IcaB protein at position I63, 226 amino acids shorter than wildtype IcaB which resulted in reversion to a non-mucoid phenotype. Lastly, two strains (#3 and #6) harboured no mutations within *ica* operon or known genes associated with staphylococcal biofilm, suggesting that alternative factors may promote loss of hyper biofilm in *S. aureus*. Epigenetic regulation has been shown to alter biofilm formation in other human pathogens such as *Haemophilus influenzae* via phase variation of the Mod DNA methyltransferase [60]. Given the lack of mutations within the *ica* operon or other biofilm regulatory genes, it is tempting to speculate that epigenetic regulation may play a role in biofilm reversion in strains #3 and #6.

Comparative genomic analyses identified the *ica* operon in >99 % of strains across a wide and genetically diverse cohort of carriage and invasive isolates. Among these there was no difference in the number of unique alleles in strains isolated from carriage or infection. While the ubiquity of *ica* genes is consistent with gene conservation and an important role for biofilm formation in staphylococci, it does not reflect an adaptation to a commensal or pathogenic lifestyle. The *icaR_A* intergenic region, which includes binding sites for multiple regulatory elements, showed the highest variability among all clonal complexes. Detailed mutational analysis of the cohort uncovered distinct nucleotide alteration patterns within the *ica* locus, evidenced by recurrent polymorphisms across isolates (Fig. 8b–c) that were associated with mucoid and reverted phenotypes. Our findings suggest that the mucoid phenotype in *S. aureus* is often lost, both *in vitro* and in clinical settings, likely as a result of an associated fitness cost.

This study enhances our fundamental understanding of how the major human pathogen *S. aureus* forms biofilms and provides evidence that mucoid formation is not restricted to pwCF as USFL039 is derived from an acute SSTI and to our knowledge, is the first published report of a hyper PIA/PNAG producing USA300 MRSA isolate.

## CRediT authorship contribution statement

**Emily Rudolph:** Writing – review & editing, Visualization, Validation, Methodology, Investigation, Formal analysis, Data curation. **Shuxian Li:** Visualization, Validation, Methodology, Investigation, Formal analysis. **Broncio Aguilar-Sanjuan:** Visualization, Validation, Software, Formal analysis, Data curation. **Seungwon Ko:** Software, Methodology, Formal analysis, Data curation. **Priyanshu S. Raikwar:** Software, Methodology, Investigation, Formal analysis, Data curation. **Carolin M. Kobras:** Writing – review & editing, Visualization, Validation, Supervision, Resources, Investigation, Formal analysis. **Serena Bettoni:** Validation, Supervision, Methodology, Investigation, Formal analysis. **Samuel K. Sheppard:** Writing – review & editing, Writing – original draft, Supervision, Resources, Project administration, Formal analysis, Conceptualization. **Maisem Laabei:** Writing – review & editing, Writing – original draft, Supervision, Resources, Project administration, Investigation, Funding acquisition, Conceptualization.

## Competing interests

All authors declare no financial or non-financial competing interests.

## Data Availability

I will shared my data via link to FigShare
