# Supplementary material for: Functional and comparative genomic characterization of biofilm formation in Staphylococcus aureus
Source: Biofilm. 2025 Dec 9;11:100341. doi: 10.1016/j.bioflm.2025.100341 (PMC12771081; doi:10.1016/j.bioflm.2025.100341)
Supplement: Multimedia component 1 [file mmc1.docx]

**Supplementary Information**

**Supplementary Table 1: List of strains and primers used in this study.**

| **Isolate / reference** | **Genotype** | **Infectious source** | **Reference or ENA accession no.** |
| --- | --- | --- | --- |
| MR005 (1) | CC8 USA300 | Blood (bacteraemia) | ERR449858 |
| MR007 (1) | CC8 USA300 | Blood (bacteraemia) | ERR449859 |
| MR018 (1) | CC8 USA300 | Blood (bacteraemia) | ERR449860 |
| MR019 (1) | CC8 USA300 | Blood (bacteraemia) | ERR449861 |
| MR021 (1) | CC8 USA300 | Blood (bacteraemia) | ERR449862 |
| MR022 (1) | CC8 USA300 | Blood (bacteraemia) | ERR449863 |
| MR023 (1) | CC8 USA300 | Blood (bacteraemia) | ERR449864 |
| MR025 (1) | CC8 USA300 | Blood (bacteraemia) | ERR449865 |
| MR026 (1) | CC8 USA300 | Blood (bacteraemia) | ERR449866 |
| MR027 (1) | CC8 USA300 | Blood (bacteraemia) | ERR449867 |
| MR029 (1) | CC8 USA300 | Blood (bacteraemia) | ERR449868 |
| MR030 (1) | CC8 USA300 | Blood (bacteraemia) | ERR449869 |
| MR031 (1) | CC8 USA300 | Blood (bacteraemia) | ERR449870 |
| MR035 (1) | CC8 USA300 | Blood (bacteraemia) | ERR449871 |
| MR036 (1) | CC8 USA300 | Blood (bacteraemia) | ERR449872 |
| MR039 (1) | CC8 USA300 | Blood (bacteraemia) | ERR449873 |
| MR047 (1) | CC8 USA300 | Blood (bacteraemia) | ERR449874 |
| MR051 (1) | CC8 USA300 | Blood (bacteraemia) | ERR449875 |
| MR060 (1) | CC8 USA300 | Blood (bacteraemia) | ERR449876 |
| MR063 (1) | CC8 USA300 | Blood (bacteraemia) | ERR449877 |
| MR064 (1) | CC8 USA300 | Blood (bacteraemia) | ERR449878 |
| MR065 (1) | CC8 USA300 | Blood (bacteraemia) | ERR449879 |
| MR072 (1) | CC8 USA300 | Blood (bacteraemia) | ERR449880 |
| MR073 (1) | CC8 USA300 | Blood (bacteraemia) | ERR449881 |
| MR074 (1) | CC8 USA300 | Blood (bacteraemia) | ERR449882 |
| MR077 (1) | CC8 USA300 | Blood (bacteraemia) | ERR449883 |
| MR078 (1) | CC8 USA300 | Blood (bacteraemia) | ERR449884 |
| MR081 (1) | CC8 USA300 | Blood (bacteraemia) | ERR449885 |
| MR083 (1) | CC8 USA300 | Blood (bacteraemia) | ERR449886 |
| MR084 (1) | CC8 USA300 | Blood (bacteraemia) | ERR449887 |
| MR087 (1) | CC8 USA300 | Blood (bacteraemia) | ERR449888 |
| MR090 (1) | CC8 USA300 | Blood (bacteraemia) | ERR449889 |
| MR091 (1) | CC8 USA300 | Blood (bacteraemia) | ERR449890 |
| MR096 (1) | CC8 USA300 | Blood (bacteraemia) | ERR449891 |
| MR107 (1) | CC8 USA300 | Blood (bacteraemia) | ERR449892 |
| MR110 (1) | CC8 USA300 | Blood (bacteraemia) | ERR449893 |
| USFL008 (2) | CC8 USA300 | Nose/skin (carriage) | ERR134787 |
| USFL009 (2) | CC8 USA300 | Nose/skin (carriage) | ERR134788 |
| USFL012 (2) | CC8 USA300 | Nose/skin (carriage) | ERR134791 |
| USFL028 (2) | CC8 USA300 | Nose/skin (carriage) | ERR134808 |
| USFL042 (2) | CC8 USA300 | Nose/skin (carriage) | ERR134822 |
| USFL061 (2) | CC8 USA300 | Nose/skin (carriage) | ERR134841 |
| USFL063 (2) | CC8 USA300 | Nose/skin (carriage) | ERR134843 |
| USFL074 (2) | CC8 USA300 | Nose/skin (carriage) | ERR134855 |
| USFL077 (2) | CC8 USA300 | Nose/skin (carriage) | ERR134858 |
| USFL082 (2) | CC8 USA300 | Nose/skin (carriage) | ERR134863 |
| USFL093 (2) | CC8 USA300 | Nose/skin (carriage) | ERR134874 |
| USFL119 (2) | CC8 USA300 | Nose/skin (carriage) | ERR302767 |
| USFL130 (2) | CC8 USA300 | Nose/skin (carriage) | ERR302778 |
| USFL141 (2) | CC8 USA300 | Nose/skin (carriage) | ERR302789 |
| USFL153 (2) | CC8 USA300 | Nose/skin (carriage) | ERR302801 |
| USFL156 (2) | CC8 USA300 | Nose/skin (carriage) | ERR302804 |
| USFL166 (2) | CC8 USA300 | Nose/skin (carriage) | ERR302815 |
| USFL167 (2) | CC8 USA300 | Nose/skin (carriage) | ERR302816 |
| USFL169 (2) | CC8 USA300 | Nose/skin (carriage) | ERR302818 |
| USFL182 (2) | CC8 USA300 | Nose/skin (carriage) | ERR302831 |
| USFL200 (2) | CC8 USA300 | Nose/skin (carriage) | ERR134623 |
| USFL211 (2) | CC8 USA300 | Nose/skin (carriage) | ERR134635 |
| USFL213 (2) | CC8 USA300 | Nose/skin (carriage) | ERR134637 |
| USFL224 (2) | CC8 USA300 | Nose/skin (carriage) | ERR134648 |
| USFL225 (2) | CC8 USA300 | Nose/skin (carriage) | ERR134649 |
| USFL230 (2) | CC8 USA300 | Nose/skin (carriage) | ERR134654 |
| USFL231 (2) | CC8 USA300 | Nose/skin (carriage) | ERR134655 |
| USFL243 (2) | CC8 USA300 | Nose/skin (carriage) | ERR134667 |
| USFL248 (2) | CC8 USA300 | Nose/skin (carriage) | ERR134672 |
| USFL259 (2) | CC8 USA300 | Nose/skin (carriage) | ERR134684 |
| USFL263 (2) | CC8 USA300 | Nose/skin (carriage) | ERR134688 |
| USFL267 (2) | CC8 USA300 | Nose/skin (carriage) | ERR134692 |
| USFL269 (2) | CC8 USA300 | Nose/skin (carriage) | ERR134694 |
| USFL271 (2) | CC8 USA300 | Nose/skin (carriage) | ERR134696 |
| USFL272 (2) | CC8 USA300 | Nose/skin (carriage) | ERR134697 |
| USFL302 (2) | CC8 USA300 | Nose/skin (carriage) | ERR134728 |
| USFL303 (2) | CC8 USA300 | Nose/skin (carriage) | ERR134729 |
| USFL304 (2) | CC8 USA300 | Nose/skin (carriage) | ERR134730 |
| USFL016 (2) | CC8 USA300 | Abscess (SSTI) | ERR134793 |
| USFL018 (2) | CC8 USA300 | Abscess (SSTI) | ERR134797 |
| USFL020 (2) | CC8 USA300 | Abscess (SSTI) | ERR134800 |
| USFL021 (2) | CC8 USA300 | Abscess (SSTI) | ERR134801 |
| USFL034 (2) | CC8 USA300 | Abscess (SSTI) | ERR134814 |
| USFL035 (2) | CC8 USA300 | Abscess (SSTI) | ERR134815 |
| USFL036 (2) | CC8 USA300 | Abscess (SSTI) | ERR134816 |
| USFL039 (2) | CC8 USA300 | Abscess (SSTI) | ERR134819 |
| USFL056 (2) | CC8 USA300 | Abscess (SSTI) | ERR134836 |
| USFL057 (2) | CC8 USA300 | Abscess (SSTI) | ERR134837 |
| USFL059 (2) | CC8 USA300 | Abscess (SSTI) | ERR134839 |
| USFL069 (2) | CC8 USA300 | Abscess (SSTI) | ERR134850 |
| USFL095 (2) | CC8 USA300 | Abscess (SSTI) | ERR302742 |
| USFL097 (2) | CC8 USA300 | Abscess (SSTI) | ERR302744 |
| USFL103 (2) | CC8 USA300 | Abscess (SSTI) | ERR302750 |
| USFL110 (2) | CC8 USA300 | Abscess (SSTI) | ERR302755 |
| USFL111 (2) | CC8 USA300 | Abscess (SSTI) | ERR302758 |
| USFL113 (2) | CC8 USA300 | Abscess (SSTI) | ERR302761 |
| USFL136 (2) | CC8 USA300 | Abscess (SSTI) | ERR302784 |
| USFL137 (2) | CC8 USA300 | Abscess (SSTI) | ERR302785 |
| USFL138 (2) | CC8 USA300 | Abscess (SSTI) | ERR223153 |
| USFL139 (2) | CC8 USA300 | Abscess (SSTI) | ERR223154 |
| USFL149 (2) | CC8 USA300 | Abscess (SSTI) | ERR302797 |
| USFL152 (2) | CC8 USA300 | Abscess (SSTI) | ERR302800 |
| USFL158 (2) | CC8 USA300 | Abscess (SSTI) | ERR302806 |
| USFL159 (2) | CC8 USA300 | Abscess (SSTI) | ERR302807 |
| USFL160 (2) | CC8 USA300 | Abscess (SSTI) | ERR302808 |
| USFL162 (2) | CC8 USA300 | Abscess (SSTI) | ERR302810 |
| USFL164 (2) | CC8 USA300 | Abscess (SSTI) | ERR302813 |
| USFL165 (2) | CC8 USA300 | Abscess (SSTI) | ERR302814 |
| USFL173 (2) | CC8 USA300 | Abscess (SSTI) | ERR302822 |
| USFL174 (2) | CC8 USA300 | Abscess (SSTI) | ERR302823 |
| USFL194 (2) | CC8 USA300 | Abscess (SSTI) | ERR134617 |
| USFL198 (2) | CC8 USA300 | Abscess (SSTI) | ERR134621 |
| USFL218 (2) | CC8 USA300 | Abscess (SSTI) | ERR134642 |
| USFL219 (2) | CC8 USA300 | Abscess (SSTI) | ERR134643 |
| USFL220 (2) | CC8 USA300 | Abscess (SSTI) | ERR134644 |
| USFL221 (2) | CC8 USA300 | Abscess (SSTI) | ERR134645 |
| USFL222 (2) | CC8 USA300 | Abscess (SSTI) | ERR134646 |
| USFL223 (2) | CC8 USA300 | Abscess (SSTI) | ERR134647 |
| USFL237 (2) | CC8 USA300 | Abscess (SSTI) | ERR134661 |
| USFL239 (2) | CC8 USA300 | Abscess (SSTI) | ERR134663 |
| USFL240 (2) | CC8 USA300 | Abscess (SSTI) | ERR134664 |
| USFL250 (2) | CC8 USA300 | Abscess (SSTI) | ERR134674 |
| USFL255 (2) | CC8 USA300 | Abscess (SSTI) | ERR134679 |
| USFL256 (2) | CC8 USA300 | Abscess (SSTI) | ERR134680 |
| USFL258 (2) | CC8 USA300 | Abscess (SSTI) | ERR134683 |
| USFL273 (2) | CC8 USA300 | Abscess (SSTI) | ERR134698 |
| USFL274 (2) | CC8 USA300 | Abscess (SSTI) | ERR134699 |
| USFL276 (2) | CC8 USA300 | Abscess (SSTI) | ERR134701 |
| USFL277 (2) | CC8 USA300 | Abscess (SSTI) | ERR134702 |
| USFL279 (2) | CC8 USA300 | Abscess (SSTI) | ERR134704 |
| USFL282 (2) | CC8 USA300 | Abscess (SSTI) | ERR134707 |
| USFL319 (2) | CC8 USA300 | Abscess (SSTI) | ERR134745 |
| USFL320 (2) | CC8 USA300 | Abscess (SSTI) | ERR134746 |
| USFL326 (2) | CC8 USA300 | Abscess (SSTI) | ERR223157 |
| USFL327 (2) | CC8 USA300 | Abscess (SSTI) | ERR223158 |
| USFL330 (2) | CC8 USA300 | Abscess (SSTI) | ERR134756 |
| USFL339 (2) | CC8 USA300 | Abscess (SSTI) | ERR134765 |
| USFL341 (2) | CC8 USA300 | Abscess (SSTI) | ERR134767 |
| USFL039nm #1 | CC8 USA300 | Abscess (SSTI); in vitro serial passage | ERR15337440 |
| USFL039nm #2 | CC8 USA300 | Abscess (SSTI); in vitro serial passage | ERR15337439 |
| USFL039nm #3 | CC8 USA300 | Abscess (SSTI); in vitro serial passage | ERR15337438 |
| USFL039nm #4 | CC8 USA300 | Abscess (SSTI); in vitro serial passage | ERR15337437 |
| USFL039nm #5 | CC8 USA300 | Abscess (SSTI); in vitro serial passage | ERR15337436 |
| USFL039nm #6 | CC8 USA300 | Abscess (SSTI); in vitro serial passage | ERR15337435 |
| USFL039nm #7 | CC8 USA300 | Abscess (SSTI); in vitro serial passage | ERR15337434 |
| USFL039nm #8 | CC8 USA300 | Abscess (SSTI); in vitro serial passage | ERR15337433 |

| **NTML / Strain ID** | **Accession Number / Description** |
| --- | --- |
| TW20 | HA-MRSA isolate, CC239 (3) |
| JE2 | Wildtype strain for the NTML (4) |
| USFL039L | Mutated USFL039 conferring low biofilm phenotype |
| NE47 | SAUSA300_1393 |
| NE156 | SAUSA300_0351 |
| NE258 | SAUSA300_2044 |
| NE437 | SAUSA300_0291 |
| NE545 | SAUSA300_0298 |
| NE558 | SAUSA300_1769 |
| NE643 | SAUSA300_0102 |
| NE704 | SAUSA300_0537 |
| NE764 | SAUSA300_1432 |
| NE787 | SAUSA300_0198 |
| NE986 | SAUSA300_2412 |
| NE1038 | SAUSA300_2610 |
| NE1128 | SAUSA300_0490 |
| NE1327 | SAUSA300_0393 |
| NE1427 | SAUSA300_1576 |
| NE1447 | SAUSA300_1470 |
| NE1474 | SAUSA300_1518 |
| NE1589 | SAUSA300_0798 |
| NE1664 | SAUSA300_2567 |
| NE1794 | SAUSA300_1575 |
| NE1840 | SAUSA300_1985 |
| NE1842 | SAUSA300_0397 |
| NE1863 | SAUSA300_0398 |
| NE1871 | SAUSA300_2284 |
| NE1882 | SAUSA300_2393 |

| **Primer** | **Sequence (5’-3’)** |
| --- | --- |
| lukE (FW) | GATTGCACCTTTAGCATCTCC |
| lukE (RV) | ACATTTTGAGTAACGCCCC |
| araB (FW) | TTACTGACGAAAACCTTAACCC |
| araB (RV) | CCCGCTTCCATAATATAAGCC |
| NE764 (FW) | CTACTGACGCATGATCTCTTC |
| NE764 (RV) | GTGTAGAGTTTTGCTTTCAGC |
| metQ1 (FW) | GATATTAAAACAATTAACGATTACAC |
| metQ1 (RV) | CAAAGTTCGAATTAATGATAACAGC |
| arcC (FW) | TCCACGATTTGATAACCCAAC |
| arcC (RV) | ATAACCGCTTCAACACCTTC |

**Supplementary Table 2: List of significant GWAS hits**

| **Gene/Unitig Name** | **hits** | **maxp** | **avg_af** | **avg_maf** | **avg_beta** | **Strain Name** | **Locus** |
| --- | --- | --- | --- | --- | --- | --- | --- |
| *alsS* | 3 | 6.124 | 0.386 | 0.386 | 0.027 | NE1794 | SAUSA300_1575 |
| *metQ1* | 7 | 5.790 | 0.545 | 0.455 | 0.048 | NE1589 | SAUSA300_0798 |
| FBONBOFM_02167 | 11 | 4.305 | 0.100 | 0.100 | 0.015 | NE764 | SAUSA300_1432 |
| NLANLCGL_02692 | 1 | 3.471 | 0.841 | 0.159 | 0.079 |  | SAUSA300_2144 |
| *yezG_3* | 3 | 3.031 | 0.617 | 0.383 | 0.017 | NE545 | SAUSA300_0298 |
| NLANLCGL_00537 | 6 | 2.812 | 0.935 | 0.065 | 0.045 | NE1863 | SAUSA300_0398 |
| NLANLCGL_00538 | 6 | 2.441 | 0.935 | 0.065 | 0.037 | NE1842 | SAUSA300_0397 |
| *lukEv* | 2 | 2.441 | 0.500 | 0.500 | 0.091 | NE558 | SAUSA300_1769 |
| *cls_2* | 5 | 2.441 | 0.587 | 0.413 | 0.036 | NE258 | SAUSA300_2044 |
| NLANLCGL_00586 | 1 | 2.360 | 0.907 | 0.093 | 0.024 |  | SAUSA300_0350 |
| IEALGAFJ_00737 | 1 | 2.360 | 0.094 | 0.094 | 0.023 | NE156 | SAUSA300_0351 |
| *recD* | 2 | 2.360 | 0.500 | 0.500 | 0.023 | NE1427 | SAUSA300_1576 |
| NLANLCGL_02323 | 1 | 2.360 | 0.907 | 0.093 | 0.022 |  | SAUSA300_0569 |
| *ltaS* | 2 | 2.360 | 0.500 | 0.500 | 0.023 |  | SAUSA300_0703 |
| IEALGAFJ_01671 | 1 | 2.360 | 0.094 | 0.094 | 0.023 | NE1871 | SAUSA300_2284 |
| IEALGAFJ_00221 | 1 | 2.360 | 0.094 | 0.094 | 0.023 | NE47 | SAUSA300_1393 |
| NLANLCGL_02438 | 8 | 2.012 | 0.959 | 0.041 | 0.077 | NE1840 | SAUSA300_1985 |
| *hisC_1* | 3 | 1.690 | 0.623 | 0.377 | 0.037 | NE1038 | SAUSA300_2610 |
| MOFEKFEK_01346 | 2 | 1.310 | 0.037 | 0.037 | 0.171 |  | SAUSA300_1727 |
| NLANLCGL_00645 | 2 | 1.269 | 0.972 | 0.028 | 0.178 | NE437 | SAUSA300_0291 |
| NLANLCGL_01629 | 2 | 1.250 | 0.935 | 0.066 | 0.149 | NE986 | SAUSA300_2412 |
| *lspA* | 1 | 1.194 | 0.467 | 0.467 | 0.054 | NE1447 | SAUSA300_1470 |
| *parE* | 4 | 0.903 | 0.500 | 0.500 | 0.033 |  | SAUSA300_1250 |
| NLANLCGL_00542 | 2 | 0.903 | 0.976 | 0.024 | 0.133 | NE1327 | SAUSA300_0393 |
| *cshB* | 3 | 0.903 | 0.657 | 0.343 | 0.057 | NE1474 | SAUSA300_1518 |
| *opuCA* | 2 | 0.873 | 0.500 | 0.500 | 0.045 | NE1882 | SAUSA300_2393 |
| NLANLCGL_00767 | 1 | 0.873 | 0.972 | 0.028 | 0.140 | NE787 | SAUSA300_0198 |
| NLANLCGL_00007 | 14 | 0.873 | 0.980 | 0.020 | 0.094 | NE643 | SAUSA300_0102 |
| *hslO* | 2 | 0.873 | 0.500 | 0.500 | 0.045 | NE1128 | SAUSA300_0490 |
| *dps* | 2 | 0.873 | 0.500 | 0.500 | 0.045 |  | SAUSA300_2092 |
| *arcC2* | 2 | 0.873 | 0.500 | 0.500 | 0.045 | NE1664 | SAUSA300_2567 |
| *araB* | 2 | 0.873 | 0.500 | 0.500 | 0.045 | NE704 | SAUSA300_0537 |

*Hits: the number of k-mer hits found to be associated with the phenotype. Maxp: the maximum p-value for k-mers mapped to the gene. Avg_maf: the average minor allele frequency of the proportion of isolates with the minor allele present. Ave_beta: the average effect size of variants mapping to the gene. Effect size: measure of the predicted contribution of the variation within the gene to the observed phenotype.*

**Supplementary Table 3:**

|  | **Minimum inhibitory concentration (μg/mL)** | |
| --- | --- | --- |
| **Antibiotic** | **LAC** | **USFL039** |
| Vancomycin | 1 | 1 |
| Teicoplanin | 0.5-2 | 1 |
| Daptomycin | 1 | 0.5 |
| Polymyxin B | 128 | 128 |
| Colistin | 256-512 | 512 |
| Imipenem | 4 | 4 |
| Nisin | 256 | 256 |
| Cefaclor | 16 | 16 |

**Supplementary Table 4: List of mutations found in USFL039 compared to reference strain LAC**

| **Position** | **Mutation Type** | **Amino Acid Change** | **Locus Tag** | **Gene Description** |
| --- | --- | --- | --- | --- |
| 41290 | Frame shift; AT>A | F22 | HUW68_RS00165 | M56 family metallopeptidase |
| 183514 | Non-synonymous; T>G | L235V | HUW68_RS00840 | Glycosyltransferase |
| 288929 | Non-synonymous; C>T | A266V | HUW68_RS01255 | BglG family transcription anti-terminator |
| 359316 | Non-synonymous; A>G | I8V | HUW68_RS01620 | ABC transporter permease |
| 381906 | Non-synonymous; G>A | R310K | HUW68_RS01720 | Lipoate-protein ligase |
| 383513 | Non-synonymous; A>C | L423V | HUW68_RS01730 | PTS ascorbate transporter subunit IIC |
| 429841 | Non-synonymous; T>G | G108D | *ahpF* | Alkyl hydroperoxide reductase subunit F |
| 453424 | Non-synonymous; A>T | K135N | HUW68_RS02105 | Superantigen-like protein SSL6 |
| 456820 | Non-synonymous; G>A | G190S | HUW68_RS02120 | Superantigen-like protein SSL9 |
| 468312 | Non-synonymous; A>T | E209D | *lpl5* | Tandem-type lipoprotein Lpl5 |
| 475219 | Non-synonymous; G>A | G73D | HUW68_RS02225 | Hypothetical protein |
| 621280 | Non-synonymous; A>T | E112V | *sdrD* | MSCRAMM family adhesin SdrD |
| 623953 | Non-synonymous; C>T | T1003I | *sdrD* | MSCRAMM family adhesin SdrD |
| 822450 | Frame shift; TG>T | L353 | HUW68_RS03945 | Glycerate kinase |
| 1150798 | Non-synonymous; T>C | Y769H | *pheT* | Phenylalanine--tRNA ligase subunit beta |
| 1199945 | Non-synonymous; A>G | E19G | HUW68_RS05860 | Cell division protein SepF |
| 1231342 | Non-synonymous; T>C | Y306H | *rsmB* | 16S rRNA (cytosine(967)-C(5))-methyltransferase RsmB |
| 1289773 | Non-synonymous; C>A | R84S | *rimP* | Ribosome maturation factor RimP |
| 1378636 | Non-synonymous; A>T | I761F | HUW68_RS06715 | Exonuclease subunit SbcC |
| 1484749 | Non-synonymous; G>A | V6253I | *ebh* | Extracellular matrix binding protein |
| 1574477 | Stop gained; G>T | G1508* | HUW68_RS07520 | Phage tail tape measure protein |
| 1609976 | Non-synonymous; G>T | R128L | HUW68_RS07790 | DUF1672 domain-containing protein |
| 1612964 | Non-synonymous; A>T | S33G | *srrA* | Two-component system response regulator SrrA |
| 1690600 | Non-synonymous; A>G | T250A | *dnaJ* | Molecular chaperone DnaJ |
| 1701051 | Non-synonymous; G>A | G485S | HUW68_RS08280 | DNA internalization-related competence protein ComEC |
| 1756279 | Non-synonymous; G>T | E269D | *obgE* | GTPase ObgE |
| 2007182 | Non-synonymous; T>A | V139D | *bcp* | Thioredoxin-dependent thiol peroxidase |
| 2399140 | Non-synonymous; T>A | F61L | *modA* | Molybdate ABC transporter substrate-binding protein |
| 2551310 | Non-synonymous; A>G | H189R | *bioA* | Adenosylmethionine--8-amino-7-oxononanoate transaminase |
| 2592141 | Non-synonymous; G>A | S101N | HUW68_RS13040 | CPBP family lipoprotein N-acylation protein LnsB |
| 2627328 | Frame shift; GT>G | K185 | *sasG* | LPXTG-anchored surface protein SasG |
| 2644022 | Frame shift; A>AT | W12 | HUW68_RS13280 | DUF2188 domain-containing protein |
| 2678269 | Non-synonymous; A>C | S161R | HUW68_RS13455 | LrgB family protein |
| 2683496 | Non-synonymous; G>A | A271T | HUW68_RS13495 | Hydroxymethylglutaryl-CoA reductase |
| 2712120 | Non-synonymous; G>A | V98M | HUW68_RS13610 | Acyltransferase family protein |
| 2739810 | Non-synonymous; C>G | P167A | HUW68_RS13780 | L-lactate dehydrogenase |
| 2802804 | Non-synonymous; A>G | N299S | *gtfB* | Accessory Sec system glycosylation chaperone GtfB |
| 2816078 | Non-synonymous; A>G | S1246G | *sasA* | Serine-rich repeat glycoprotein adhesin SasA |
| 2827765 | Frame shift; TC>T | E105 | *icaR* | *ica* operon transcriptional regulator IcaR |
| 2844003 | Frame shift; AT>A | I69 | HUW68_RS14230 | GNAT family N-acetyltransferase |

Locus tag is mapped to the *S. aureus* genome NZ_CP055225

**Supplementary Table 5: List of mutations found in USFL039nm variants compared to USFL039m**

|  | | | **USFL039nm variants (1-8)** | | | | | | | |
| --- | --- | --- | --- | --- | --- | --- | --- | --- | --- | --- |
| **Position** | **Mutation** | **Gene Description** | **#1** | **#2** | **#3** | **#4** | **#5** | **#6** | **#7** | **#8** |
| 242428 | Frameshift; 204delA; A69 | IS6 family transposase IS431R | . | C | C | . | C | . | C | C |
| 242582 | Synonymous; A17A | IS6 family transposase IS431R | G | G | G | G | G | G | G | G |
| 282248 | Synonymous; L58L | IS6 family transposase IS431mec | G | G | G | G | G | G | G | G |
| 282656 | Synonymous; E194E | IS6 family transposase IS431mec | . | . | . | . | . | . | . | A |
| 361560 | Frameshift; 857_860delTATT; L286 | putative poly-beta-1,6-N-acetyl-D-glucosamine export protein (IcaC) | T | TAATA | . | . | . | . | . | T |
| 361845 | In-frame deletion; 564_575delATTCGGATGGAT; F189-Ile192 | putative poly-beta-1,6-N-acetyl-D-glucosamine export protein (IcaC) | . | . | . | . | A | . | . | . |
| 362147 | Non-synonymous; 274G>A; G92R | putative poly-beta-1,6-N-acetyl-D-glucosamine export protein (IcaC) | . | . | . | . | . | . | T | . |
| 363088 | Frameshift; 187_192delATTTAC; I63 | Poly-beta-1,6-N-acetyl-D-glucosamine N-deacetylase (IcaB) | . | . | . | ATTA | . | . | . | . |
| 202488 | Non-synonymous; 490G>T; A164S | Putative 2-hydroxyacid dehydrogenase SA2098 | A | . | . | . | . | . | . | . |
| 16419 | Non-synonymous; 14A>T; Y5F | hypothetical protein | . | . | A | . | . | . | . | . |
| 3127 | Non-synonymous; 127G>A; E43K | hypothetical protein | . | . | . | T | . | . | . | . |

**Supplementary Figure 1**


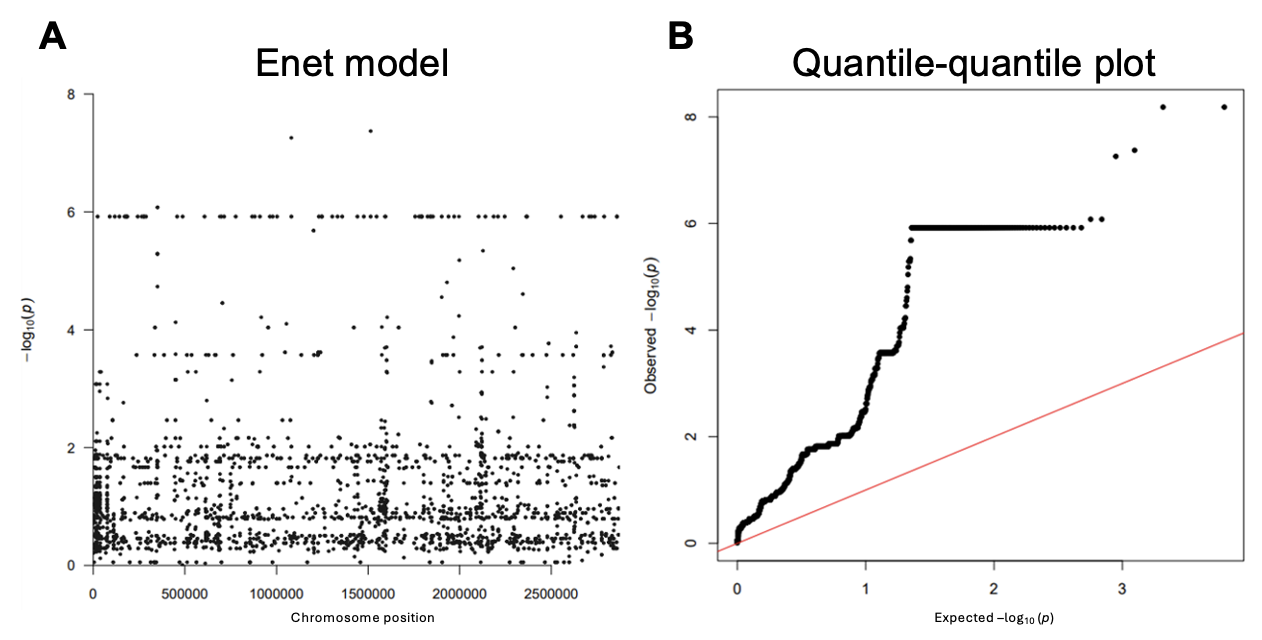


**Suppl. Fig 1:** **Genome-wide association study analysis of mutations linked to biofilm formation**. **A)** Manhattan plot displaying the association of *k*-mers with biofilm formation using an elastic net regression model and **B)** The QQ-plot of the observed p-values showing the presence of lineage effect.

**Supplementary Figure 2**

**
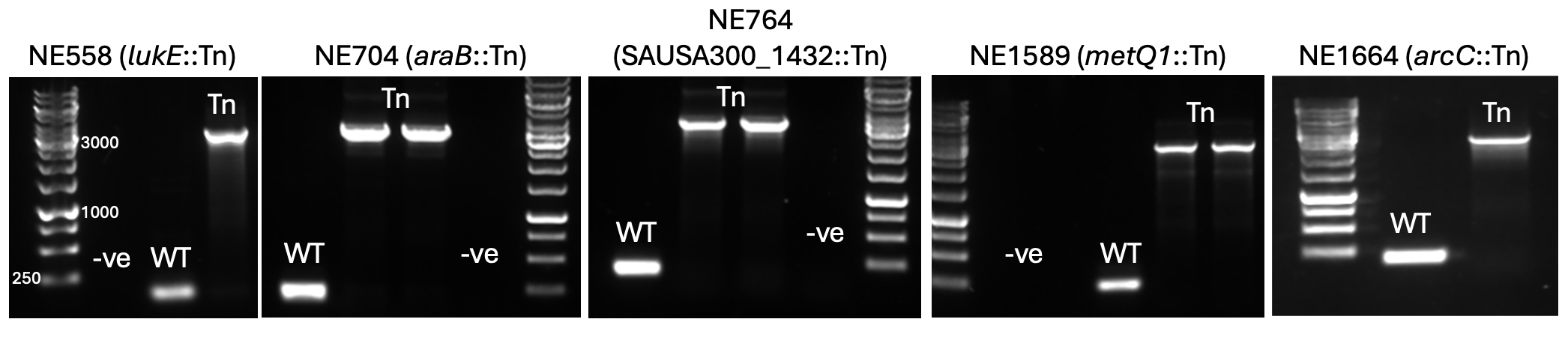
**

**Suppl. Fig 2: PCR confirmation of transposon insertion mutants.** Gene-specific primers were used to PCR amplify a region flanking the transposon (Tn) insertion site which is indicated by a band size of approx. 250-300 bp in the WT (strain JE2). Tn mutants are confirmed to contain the *bursa aurealis* transposon cassette with band size of approx. 3.5 kb (note, Tn cassette = 3.2 kb).

**Supplementary Figure 3**


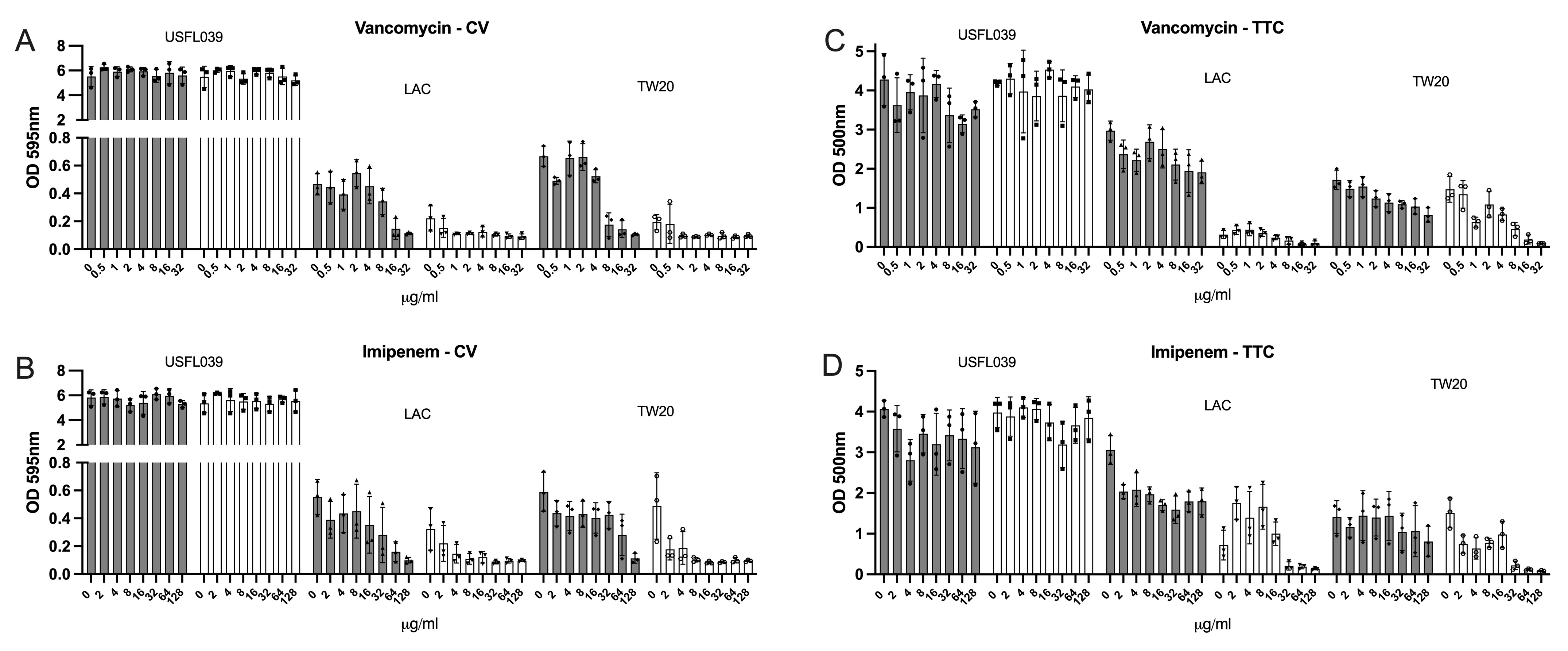


**Suppl. Fig 3: Antibiotic challenge of mature *S. aureus* biofilms.** USFL039, LAC and TW20 biofilms were allowed to mature for 24h grown in either TSB-G (dark bars) or TSB-NaCl (white bars). Biofilms were washed to remove non-adherent cells and then incubated in doubling concentrations of vancomycin (0-32 μg/ml) or imipenem (0-128 μg/ml) in respective TSB broth for 24h. **A-B**) Biomass analysis by crystal violet (CV) staining and **C-D)** metabolic activity by 2,3,5-triphenyl-tetrazolium chloride (TCC) staining was performed. Three technical replicates and three biological replicates (individual points) are shown with the error bars depicting the SD.

**Supplementary Figure 4**

**
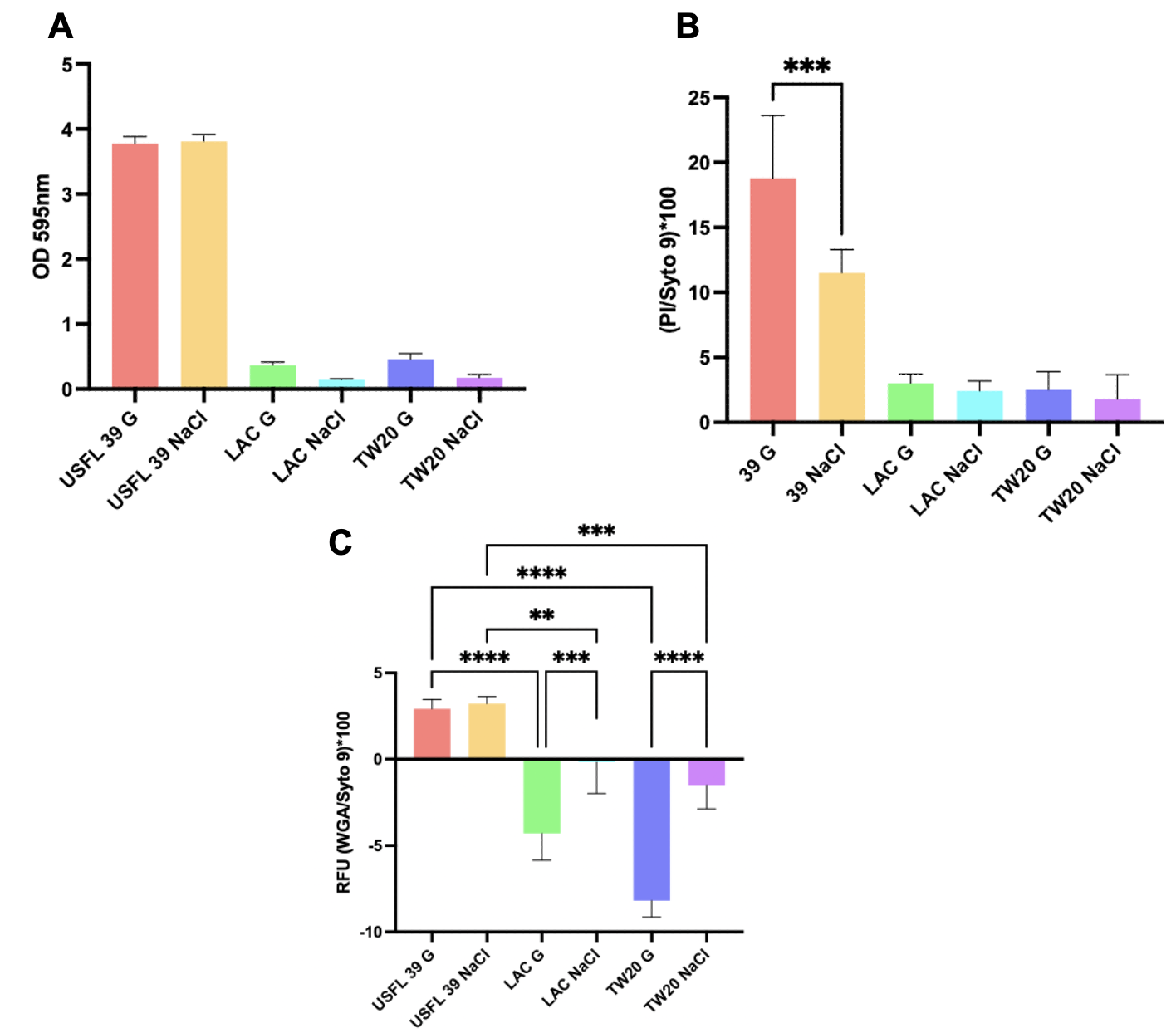
**

**Suppl. Fig 4: Biomass and live/dead staining for microscopic analysis of biofilm formation.** Cultures were grown in TSB-G or TSB-NaCl and inoculated into delta-treated 96 well plates in duplicate and incubated for 12 hours. **A)** Isolates were grown in a clear sterile delta-treated 96 well plate, all non-adhered cells were removed by washing, and the biomass was determined by crystal violet staining. **B)** Relative fluorescence was measured on a FLUOstar Omega microplate reader (BMG Labtech) at 500 nm for SYTO9 and 575 nm for propidium iodide (PI). To determine overall cell death in the biofilms, a ratio of PI and Syto 9 was measured. **C)** To determine overall PIA/PNAG presence the ratio of wheat germ agglutinin (WGA) to SYTO9 was measured. Three biological repeats were included. Statistical differences were calculated by one-way ANOVA. ***p*< 0.01. ****p*< 0.001, **** *p*< 0.0001.

**References:**

1. Laabei M, Uhlemann AC, Lowy FD, Austin ED, Yokoyama M, Ouadi K, et al. Evolutionary Trade-Offs Underlie the Multi-faceted Virulence of Staphylococcus aureus. PLoS Biol. 2015;13(9):e1002229.

2. Uhlemann AC, Dordel J, Knox JR, Raven KE, Parkhill J, Holden MT, et al. Molecular tracing of the emergence, diversification, and transmission of S. aureus sequence type 8 in a New York community. Proc Natl Acad Sci U S A. 2014;111(18):6738-43.

3. Holden MT, Lindsay JA, Corton C, Quail MA, Cockfield JD, Pathak S, et al. Genome sequence of a recently emerged, highly transmissible, multi-antibiotic- and antiseptic-resistant variant of methicillin-resistant Staphylococcus aureus, sequence type 239 (TW). J Bacteriol. 2010;192(3):888-92.

4. Fey PD, Endres JL, Yajjala VK, Widhelm TJ, Boissy RJ, Bose JL, et al. A genetic resource for rapid and comprehensive phenotype screening of nonessential Staphylococcus aureus genes. mBio. 2013;4(1):e00537-12.
